# Clinical and Pathological Findings in Coastal Birds Affected by Domoic Acid Toxicity

**DOI:** 10.3390/toxins18090401

**Published:** 2026-09-20

**Authors:** Corinne M. Gibble, Melissa A. Miller, Raphael M. Kudela, Angelina Reed, Kevin Keel, Rebecca S. Duerr

**Affiliations:** 1 California Department of Fish and Wildlife, Office of Spill Prevention and Response, Marine Wildlife Veterinary Care and Research Center, Santa Cruz, CA 95060, USA; melissa.miller@wildlife.ca.gov (M.A.M.);; 2 Department of Ocean Sciences, University of California, Santa Cruz, CA 95064, USA; 3 School of Veterinary Medicine, University of California, Davis, Davis, CA 95616, USA; 4 International Bird Rescue, Fairfield, CA 94534, USA; rebecca.duerr@birdrescue.org

**Keywords:** DA, HABs, harmful algal blooms, avian health, wildlife pathology, *Pseudo-nitzschia*, loons

## Abstract

In 2017, an unusually large number of live stranded and dead marine birds were found in California, coincident with a large bloom of the diatom *Pseudo-nitzschia* that produced the toxin domoic acid (DA). The most abundant bird species affected by this large die-off and stranding event were Pacific Loons (*Gavia pacifica*) and Red-throated Loons (*Gavia stellata*). International Bird Rescue (IBR; San Pedro, CA) received 117 live loons with suspected DA toxicosis. Many birds that survived the first few days in rehabilitation quickly re-attained suitable health metrics; however, 68 loons (58.1%) died or were considered unreleasable due to ongoing abnormal behavior or injuries that necessitated humane euthanasia. In this descriptive study, both live stranded euthanized birds and dead stranded birds were examined to characterize and better understand the effects of DA toxicosis in the avian brain and body. Utilizing a suite of diagnostics, our analysis revealed novel pathological patterns that divided these birds into three groups: those suspected of having acute, subacute, and chronic DA toxicosis. Domoic Acid was found in all groups, and the severity of the microscopic lesions appeared to correspond with the amount of time each bird survived post-stranding. Our investigation is the first known account identifying the pathological progression from acute to chronic DA toxicosis in birds.

## 1. Introduction

Harmful algal blooms (HABs) occur globally in both marine and freshwater systems, and the prevalence of toxic events is rapidly increasing in duration, magnitude, and frequency [1,2,3]. Intensifying bloom events have been predominantly associated with a warming climate, ecosystem degradation, and eutrophication, and these environmental perturbations are expected to increase and amplify over time [4,5,6]. Due to their unique life history traits, seabirds may be especially susceptible to marine HAB exposure, toxin bioaccumulation, and sustained tissue damage; these traits include central place foraging, longer life spans, late age at first reproduction, and small clutch size [7,8]. 

The neurotoxin domoic acid (DA), produced by the widespread chain-forming diatom *Pseudo-nitzschia,* has long been problematic for marine wildlife and humans. *Pseudo-nitzschia* generally blooms in the spring along the Pacific coast following nutrient surges provided by spring upwelling [9,10], and DA can transfer trophically and persist in sediment [11,12,13,14]. Published reports of exposure in seabirds are few and consist mainly of descriptions of DA-associated behavioral abnormalities and biochemical testing of DA content in tissues, gastrointestinal (GI) content, and prey species. While the gross and microscopic pathology and pathophysiology of DA is well-defined in marine mammals [15,16,17,18,19,20,21], gross and microscopic lesions associated with DA exposure are poorly defined in birds, including aquatic species such as loons that are frequently exposed to DA in their environment. 

Reported clinical signs of DA toxicosis in birds include gastroenteritis, seizures, ataxia, tremors, coma, and death [7,8]. Although neurological abnormalities are frequently described in birds during bloom conditions [22,23,24,25], documentation of the presence and concentration of DA in tissues, ingesta, digesta, and feces of affected birds is uncommon, and descriptions of DA-associated avian gross and microscopic lesions are scarce and relatively nonspecific. 

Silvagni [26] experimentally exposed Pigeons (*Columba livia*; ROPI), Mallards (*Anas platyrhynchos*; MALL), and Common Murres (*Uria aalge*; COMU) intracoelomically to DA. Diverse behavioral changes were noted, including agitation, increased ambulation, altered social interactions (MALL), loss of fear response (ROPI and MALL), depression, obtundation, chronic thermoregulatory insufficiency, and abducted wing posture (COMU). However, minimal gross and/or microscopic lesions were reported following a single dose of intracoelomic DA administration with a short (8 h) post-exposure interval which was followed by euthanasia. Although the short time interval between toxin exposure and euthanasia in this study did not allow for assessment of long-term gross and microscopic lesions associated with DA exposure, this work provided the first estimates of LD50 (Median Lethal Dose) and ED50 (Median Effective Dose) for DA in laboratory-exposed birds. The dose response varied by species, with LD50 of 900, 4000, and 4100 ng g^−1^ for ROPI, MALL, and COMU, respectively. No other accounts of experimental dosing of birds with DA have been published, and the gross and histologic lesions associated with DA toxicosis in birds are largely unknown. This creates diagnostic challenges for making post-mortem diagnoses of DA toxicosis.

During the spring of 2017, a large bloom of the diatom *Pseudo-nitzschia* occurred with high DA toxin production that coincided both temporally and spatially with a mass stranding of loons along the southern California coast. From 18 April through 1 May 2017, over 700 loons, primarily Pacific Loons (*Gavia pacifica*; PALO) and Red-throated Loons (*G. stellata*; RTLO), were found beachcast in Santa Barbara, Ventura, and Los Angeles Counties. Beach surveys reported unusually high carcass deposition (one local beach had an average of 242 avian carcasses per mile of shoreline) and some live-stranded birds exhibited seizures, head swaying, and hypersalivation [27]. Bloom conditions and associated toxin production were so severe that recreational shellfish harvest for human consumption was closed in Santa Barbara County from mid-April through late October [28].

This mass-stranding event presented an opportunity to carefully investigate clinical signs, toxicology, and lesions associated with DA toxicosis in birds by examining these wild loons naturally exposed to DA that were found freshly dead during the stranding event as well as those surviving up to 86 days post-stranding. This is the first study to assess DA toxicosis-associated clinical signs, toxicology, and histologic pathology in wild loons naturally exposed to DA with fatal acute or sublethal subacute to chronic DA toxicosis. 

## 2. Results

### 2.1. Live Bird Recovery, Hospitalization, Clinical Assessment, and Treatment

A total of 117 live loons were received for care at IBR during the 2017 mass-stranding event, including nine Common Loons (*Gavia immer*; COLO), 25 RTLO and 83 PALO. All loons were routinely dewormed orally once with 0.4 mg/kg Ivermectin and 20 mg/kg Praziquantel. Thirteen birds (11.1%) were euthanized during the first 24 h due to stranding-associated orthopedic or ocular injuries, or due to failure to respond to thermal and nutritional support, rehydration, and injury management. At admission, many loons (108/117; 92.3%) appeared dazed and abnormally calm, with nine birds (eight PALO and one RTLO) exhibiting seizures, tremors, and head swaying. These nine birds were treated with oral Diazepam (0.5–2 mg/kg intravenously or orally) or Midazolam (0.5–2 mg/kg intramuscularly or orally) as needed to extinguish overt neurological clinical signs. Seven of the nine loons with neurological clinical signs survived for more than 24 h and also received oral Phenobarbital (4 mg/kg twice daily for up to 3 days). Two of the nine loons recovered sufficiently to be released. Although hypersalivation, emesis, or regurgitation were reported for some loons at the time of field stranding recovery, these clinical signs were not reported for the birds during initial care. 

Many of the birds surviving more than 24 h quickly recovered their waterproof plumage, nutritional condition, and blood values, with 49/117 loons (41.9%) later released. Birds that were released spent between 2 days and 100 days in care (median time in care = 18 days) until behavior, body weight, and other physiological parameters were determined to be within normal limits. The remaining 68 birds (58.1%) never fully recovered and either died or were humanely euthanized due to chronic behavioral abnormalities (described below) or respiratory, foot, or leg lesions (e.g., presumptive aspergillosis airsacculitis, osteomyelitis, or toe tip necrosis). Several of the birds were unusually unresponsive to visual stimuli (e.g., minimal reaction to the approach of a net during capture from the pool; Supplementary Materials Video S1) but were hyperreactive to physical manipulation, as if startled by being touched. None of the birds were diagnosed with ocular trauma (a few had mild conjunctivitis), although detailed ophthalmologic examinations were not performed. Two of the loons were abnormally aggressive, with one alternating between hyper-aggression and obtundation; this bird was ultimately euthanized due to avian poxviral infection in order to minimize the risk of viral spread to other birds. The other hyperaggressive bird was released after 2 days of captive care because it posed a severe hazard to other birds, a private pool was not available to meet its needs, and it met all physical criteria for release (excellent nutritional condition, waterproof plumage, hematological packed cell volume and total solids within normal limits, and no respiratory abnormalities identified via radiographs or auscultation).

As the length of time in care progressed, some loons appeared to be physically healthy but neurologically abnormal; affected birds floated aimlessly and exhibited a head-tucked posture suggestive of somnolence as well as reduced avoidance of humans, which made them easy to catch and restrain. Although these birds reacted adversely to handling, abnormally low situational awareness and fear responses resumed as soon as handling ceased. Under captive conditions, these birds could maintain waterproof plumage, find food dishes, and consume fish sufficient to maintain a reasonable nutritional condition. However, due to their markedly reduced situational awareness and diminished fear responses, birds exhibiting these chronic behavioral deficits were ultimately deemed unfit for release and were humanely euthanized. 

### 2.2. Acute DA Toxicosis Suspects (0 Days to <48 h Post-Stranding; n = 10)

**Figure 1:**
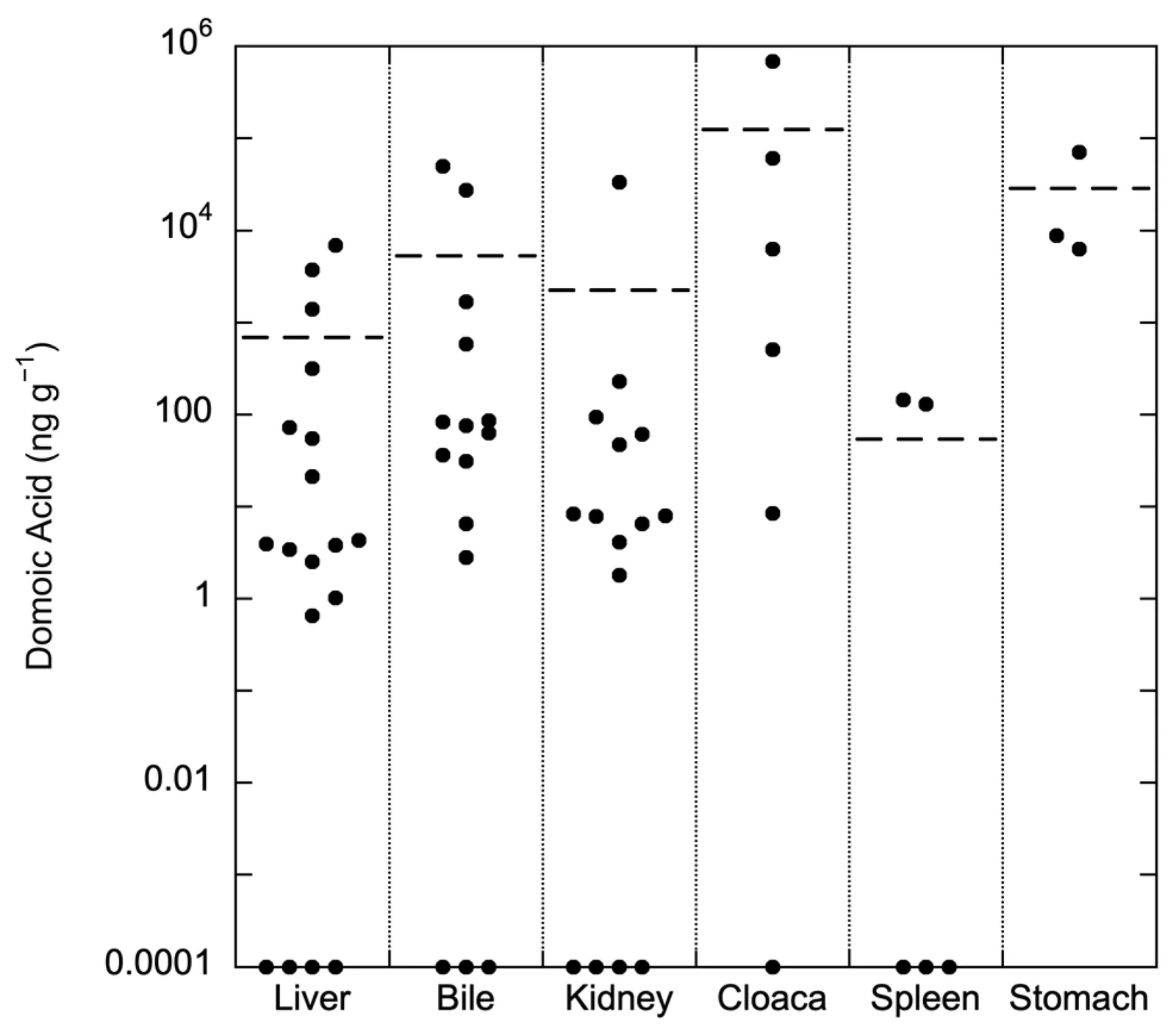
DA in organ samples of loons impacted by the 2017 mortality event. Mean values are represented by horizontal dashed lines and include zero values. DA (ng g^−1^) is plotted on a log scale.

Birds in the acute DA toxicosis suspects subgroup were found dead on beaches, died during transport to rehabilitative care and were deemed dead on arrival (DOA), or died within 48 h in rehabilitative care. Gross and microscopic findings (where available) as well as DA biochemical test results are summarized in Table S1 and Figure 1. Limited behavioral data (*n* = 2) were available for this group due to the circumstances of death before intake to rehabilitative care. In comparison, live stranded loons from the same area that died within 48 h after capture were noted to have exhibited hypersalivation, abundant peri-oral froth, obtundation, and seizures [27].

#### 2.2.1. Acute DA Toxicosis Suspects Gross Necropsy Findings (*n* = 10)

Ten loons from the event in the acute DA toxicosis subgroup that were found freshly dead and minimally decomposed were submitted to MWVCRC for necropsy; five (50%) were female, five (50%) were male, and all 10 (100%) were adults. Many had partially digested small fish in their GI tracts, which indicated that they had eaten within hours before death. 

**Figure 2:**
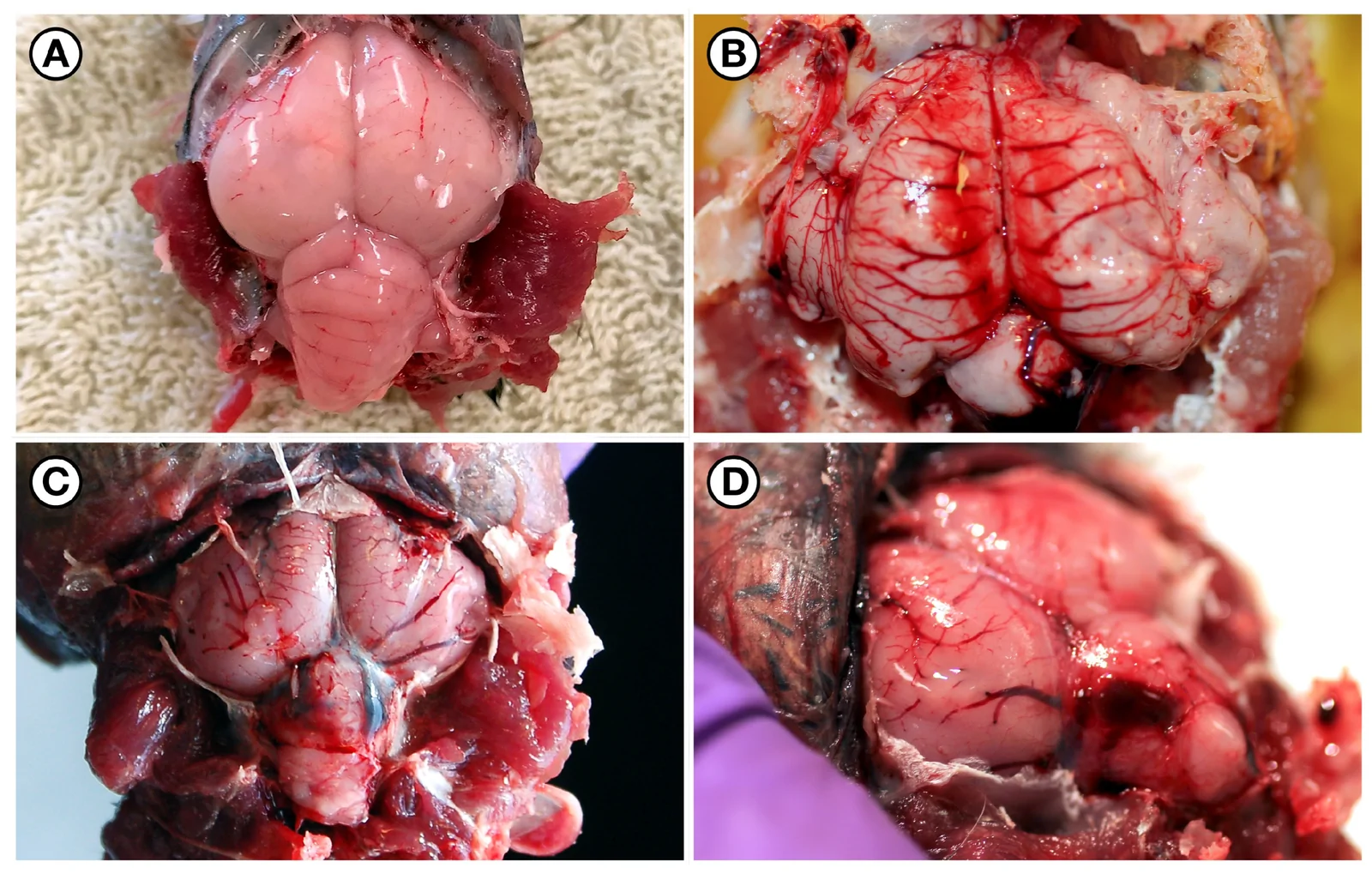
Diffuse moderate to marked vascular congestion and venous engorgement was common in birds with acute fatal DA toxicosis. (**A**) Normal brain from a negative control COLO (IBR 23-1238; shown for reference). (**B**) Brain from a BRPE (15-0100; *Pelecanus occidentalis*) that died due to acute fatal DA toxicosis (positive control). Severe diffuse brain and meningeal congestion and possible bilaterally symmetrical meningeal hemorrhage were visible along the dorsomedial aspect of both cerebral hemispheres, along with focal venous engorgement of the dorsal cerebellum. (**C**) Brain from a PALO (17-0086) that died due to acute fatal DA toxicosis. Severe diffuse brain and meningeal congestion were accompanied by cerebellar venous engorgement. (**D**) Severe diffuse brain and meningeal congestion and focal venous engorgement in another RTLO (17-0085) with acute, fatal DA toxicosis.

**Figure 3:**
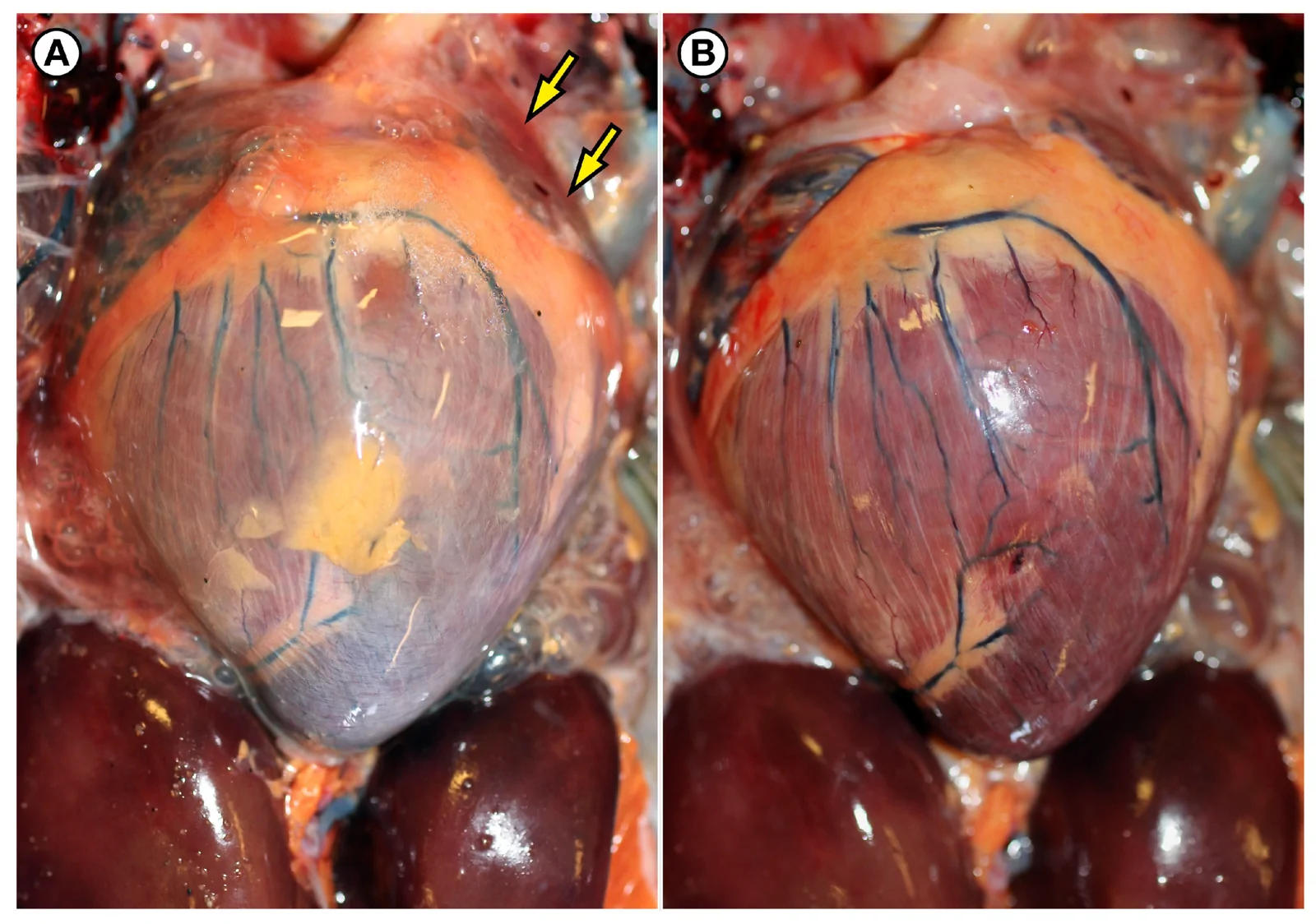
Similarly to DA-exposed marine mammals, birds dying from acute, fatal DA toxicosis presented with subtle cardiovascular pathology. (**A**) Heart from a BRPE (15-0100) that was found dead with serous pericardial effusion, characterized by mild distension of the pericardial sac with red-tinged, serous fluid (arrows) that contained 11,000 ng g^−1^ DA. (**B**) Heart from the same BRPE (15-0100) after the pericardial sac was removed. The ventricular myocardium was slightly pale and congested, and the heart was mildly distended with blood.

**Figure 4:**
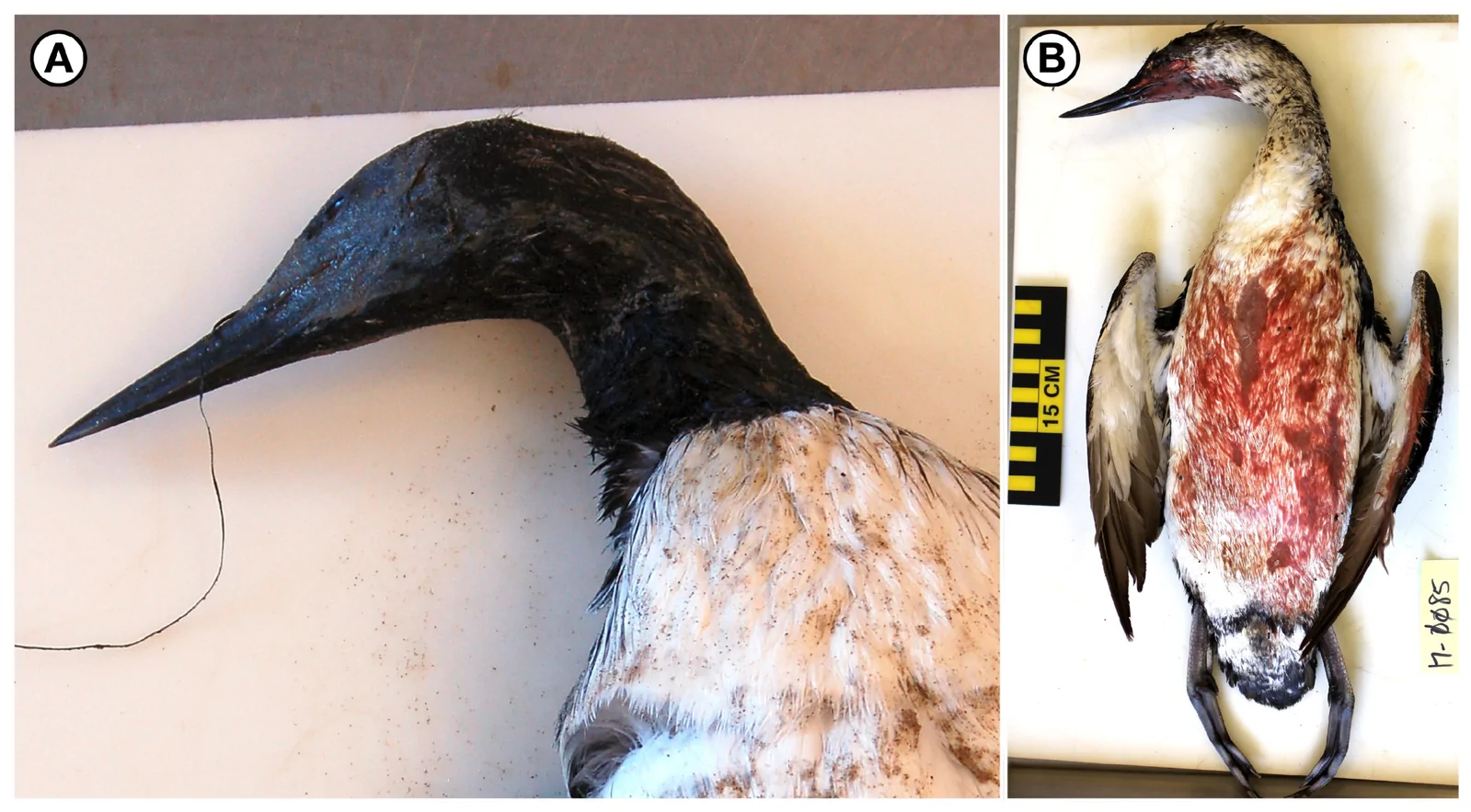
Regurgitation, hypersalivation, or potential enhanced respiratory secretions were common in birds with acute fatal DA toxicosis. (**A**) PALO (17-0086) found dead during a mass-mortality event with very high concentrations of DA in the GI content, tissues and body fluids. Dried digesta coated feathers around the mouth and down the neck. (**B**) RTLO (17-0085) found dead during a mass-mortality event with very high concentrations of DA in the GI content, tissues and body fluids. Bloody fluid coated feathers around the mouth, neck, and ventrum.

Features of gross lesions in the suspect acute DA toxicosis group (Table S1), as compared to negative and positive controls (Figure 2A,B), included severe diffuse vascular congestion and multifocal acute hemorrhage that was especially prominent in the brain (Figure 2C,D), lungs, heart, liver, adrenal glands, and kidneys. Diffuse vascular congestion was often accompanied by mild to moderate diffuse venous and cardiac engorgement, ventricular pallor, and serous pericardial effusion (Figure 3). Also noted was severe diffuse pulmonary edema characterized by markedly congested and wet lungs with abundant white froth. Two loons in the acute DA toxicosis group exhibited peri-oral feather staining suggestive of emesis, regurgitation, hypersalivation, perimortem hemorrhage, and/or respiratory hypersecretion (Figure 4). One loon exhibited diffuse pectoral muscle congestion, and another had acute hepatic and renal fragmentation with hemorrhage suggestive of perimortem blunt trauma. 

#### 2.2.2. Acute DA Toxicosis Suspects Biochemical Testing (*n* = 10)

Tissues and GI content from 10 loons in the acute DA toxicosis group were biochemically tested at UCSC for the presence and concentration of DA. DA was detected via LC/MS in the GI and cloacal content, tissues, and/or body fluids from 10/10 (100%) loons, in many cases, with extremely high toxin concentrations (Table S1). Partially digested small fish (determined to be Northern Anchovy, *Engraulis mordax*, via otolith enumeration and visual identification) were recovered from three of these birds at necropsy. A partially intact Northern Anchovy was found in the esophagus of one of the birds (17-0086); the viscera of this fish contained 88,630 ng g^−1^ DA. Peak concentrations of other GI samples for the acute DA subgroup birds were 681,190 ng g^−1^, and 71,150 ng g^−1^ DA in cloacal, esophageal, and gastric content, respectively (Table S1). Peak DA concentrations for tissues outside of the GI tract were 49,690 ng g^−1^, 33,446.55 ng g^−1^, and 6850 ng g^−1^ in bile, kidney, and liver, respectively. 

#### 2.2.3. Acute DA Toxicosis Suspects Histopathology (*n* = 3)

**Figure 5:**
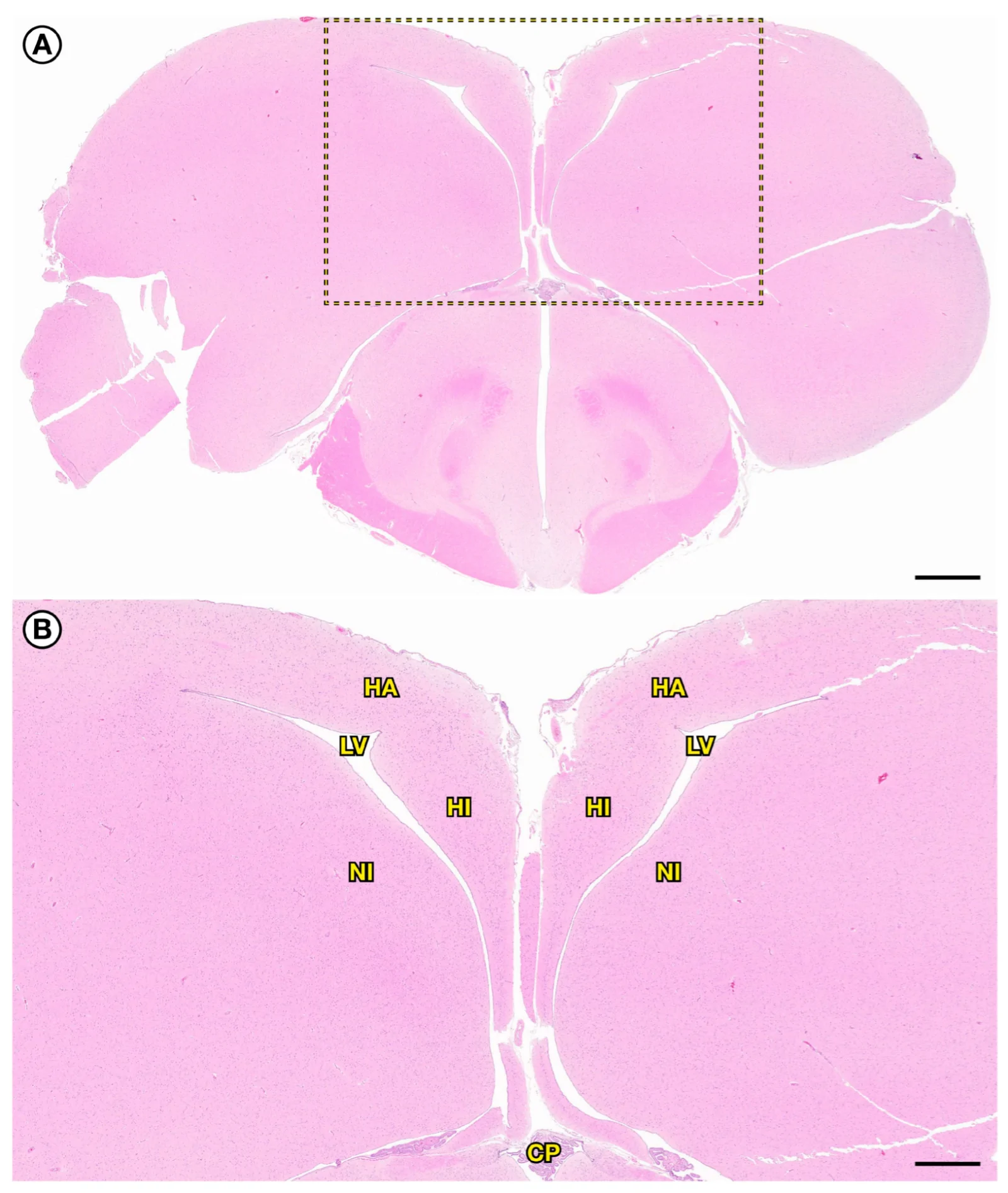
Transverse sections through the caudal cerebrum of a normal (negative control) loon. (**A**) Low-magnification view of a transverse section of normal COLO (IBR 23-1238) brain at the level of the caudal cerebrum. The cerebrum was diffusely pink and homogenous, the lateral ventricles were small and narrow, and the brain, meninges, and choroid plexus were not significantly congested. (**B**) (IBR 23-1238) Higher-magnification view of the caudodorsal cerebral midline delineated by a box in (**A**) above. Locations of the hippocampus (HI), hyperpallium apicale (HA), dorsal nidopallium (NI), lateral ventricles (LV), and choroid plexus (CP) are shown. All slides were stained with hematoxylin and eosin, with images obtained using a digital scanning microscope. Bars = 1750 and 900 µm, respectively.

**Figure 6:**
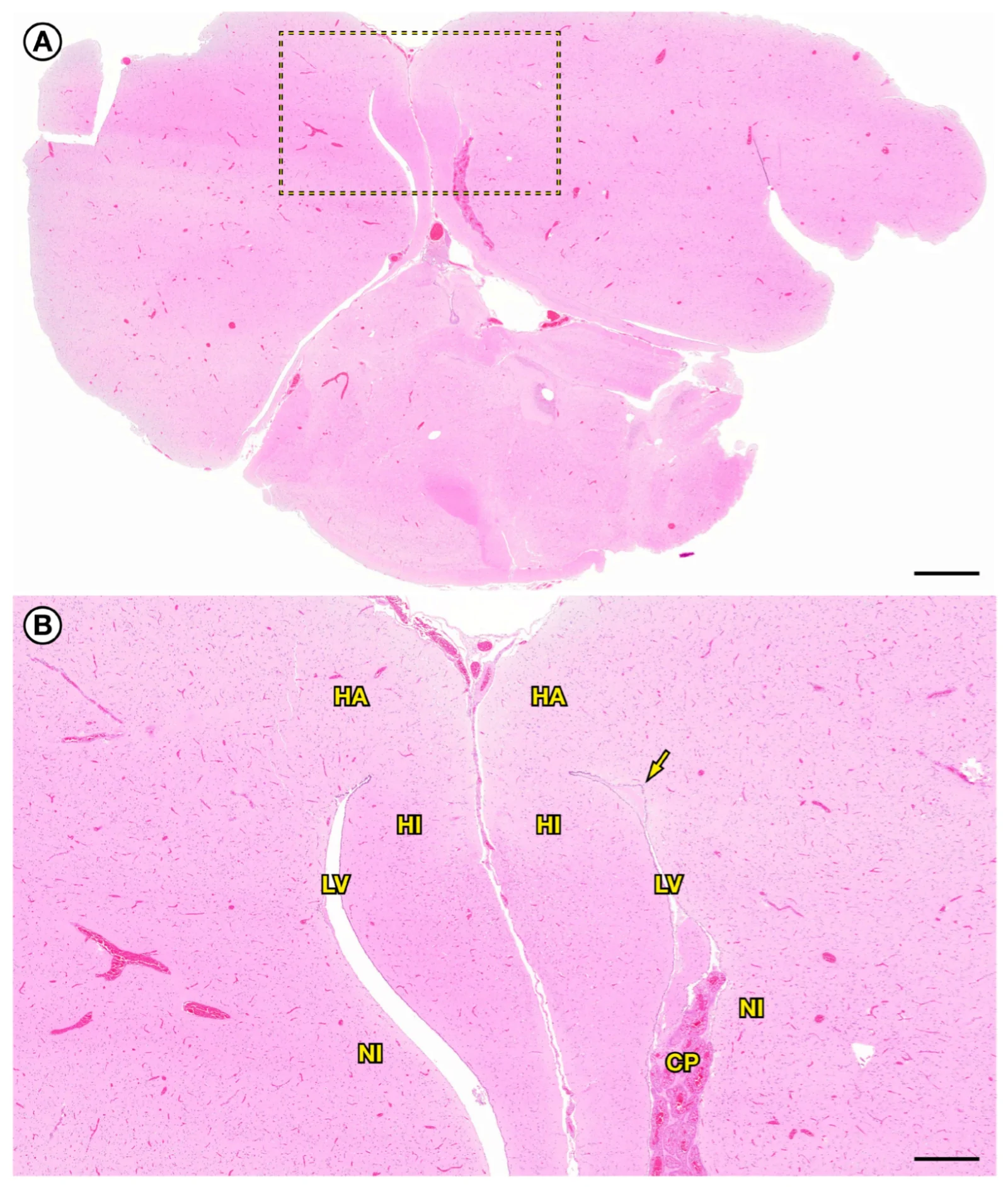
Transverse section through the caudal cerebrum of a loon that died due to acute, fatal DA toxicosis. (**A**) Low-magnification view of caudal cerebrum from a PALO (17-0086) where the cerebrum and meninges were diffusely congested. (**B**) (17-0086) Higher-magnification view of the caudodorsal cerebral midline shown within the box in (**A**) above, including the hippocampus (HI), hyperpallium apicale (HA), and dorsal nidopallium (NI). In contrast with the negative control brain shown in Figure 5A,B, the meninges, brain tissue, and choroid plexus (CP) were diffusely and markedly congested. Although the lateral ventricles (LV) were not significantly enlarged, accumulation of scant proteinacious fluid was visible (arrow at upper right). All slides were stained with hematoxylin and eosin, with images obtained using a digital scanning microscope. Bars = 1500 and 500 µm, respectively.

**Figure 7:**
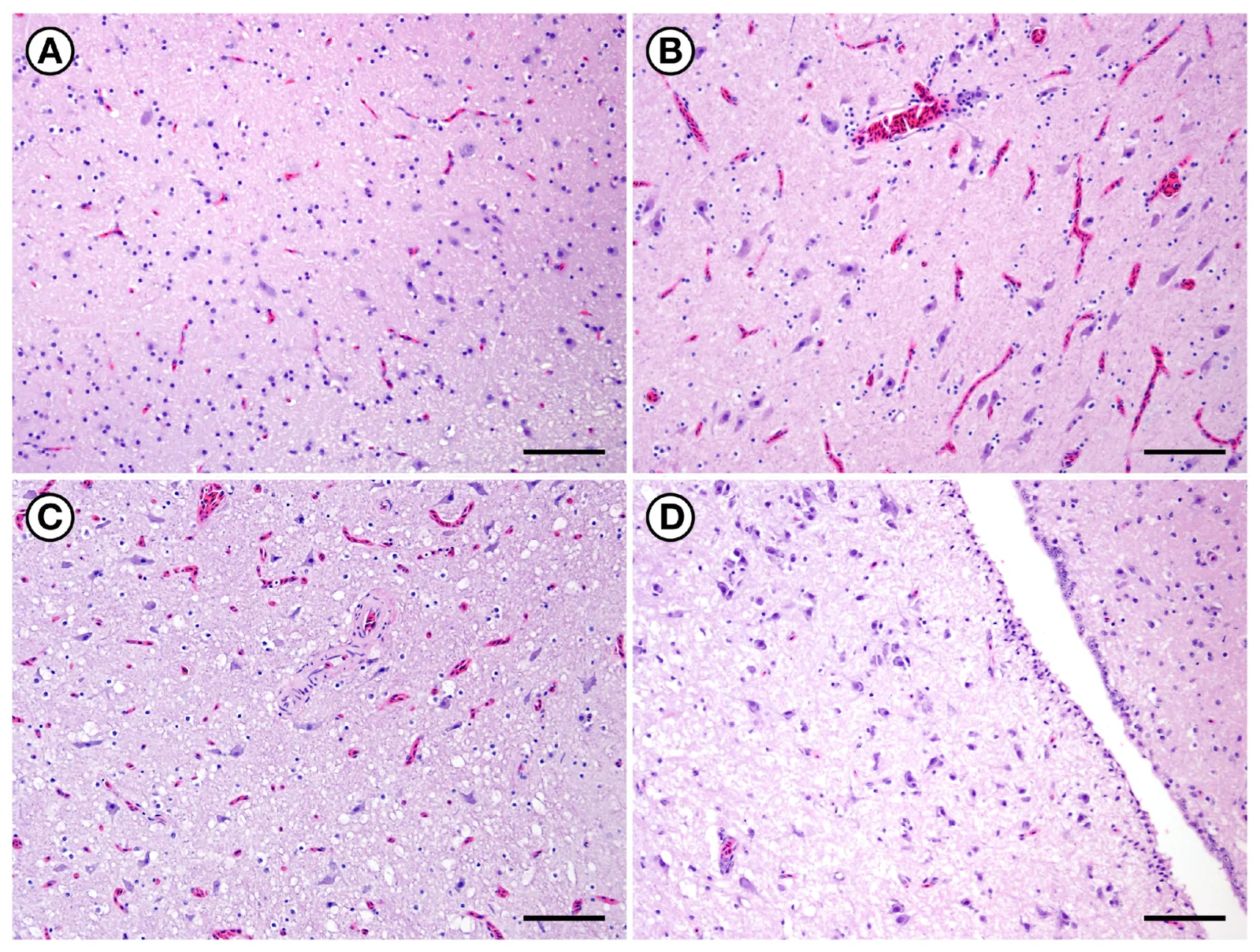
Severe vascular congestion and spongiosis were common in the brains of seabirds with DA toxicosis: (**A**) Reference image of negative control PALO (06-0219) brain tissue for comparison. (**B**) Brain from positive control BRPE (15-0100); postmortem tissues and GI content from this bird had DA concentrations as high as 75,300 PPB. There was severe diffuse vascular congestion and mild spongiosis. (**C**) Brain from PALO (17-0086) with acute, fatal DA toxicosis; postmortem tissues and GI content had DA concentrations as high as 681,190 PPB. There was moderate to marked diffuse vascular congestion and spongiosis. (**D**) Brain from PALO (IBR 17-0406) that was euthanized after 17 days of captive care due to chronic behavioral abnormalities; postmortem cloacal content was strongly DA-positive (504 PPB). Brain histopathology was characterized by patchy spongiosis without the diffuse marked congestion that was characteristic of birds that died acutely during the DA event. All slides were stained with hematoxylin and eosin. Bars = 75, 38, 75, and 75 µm, respectively.

**Figure 8:**
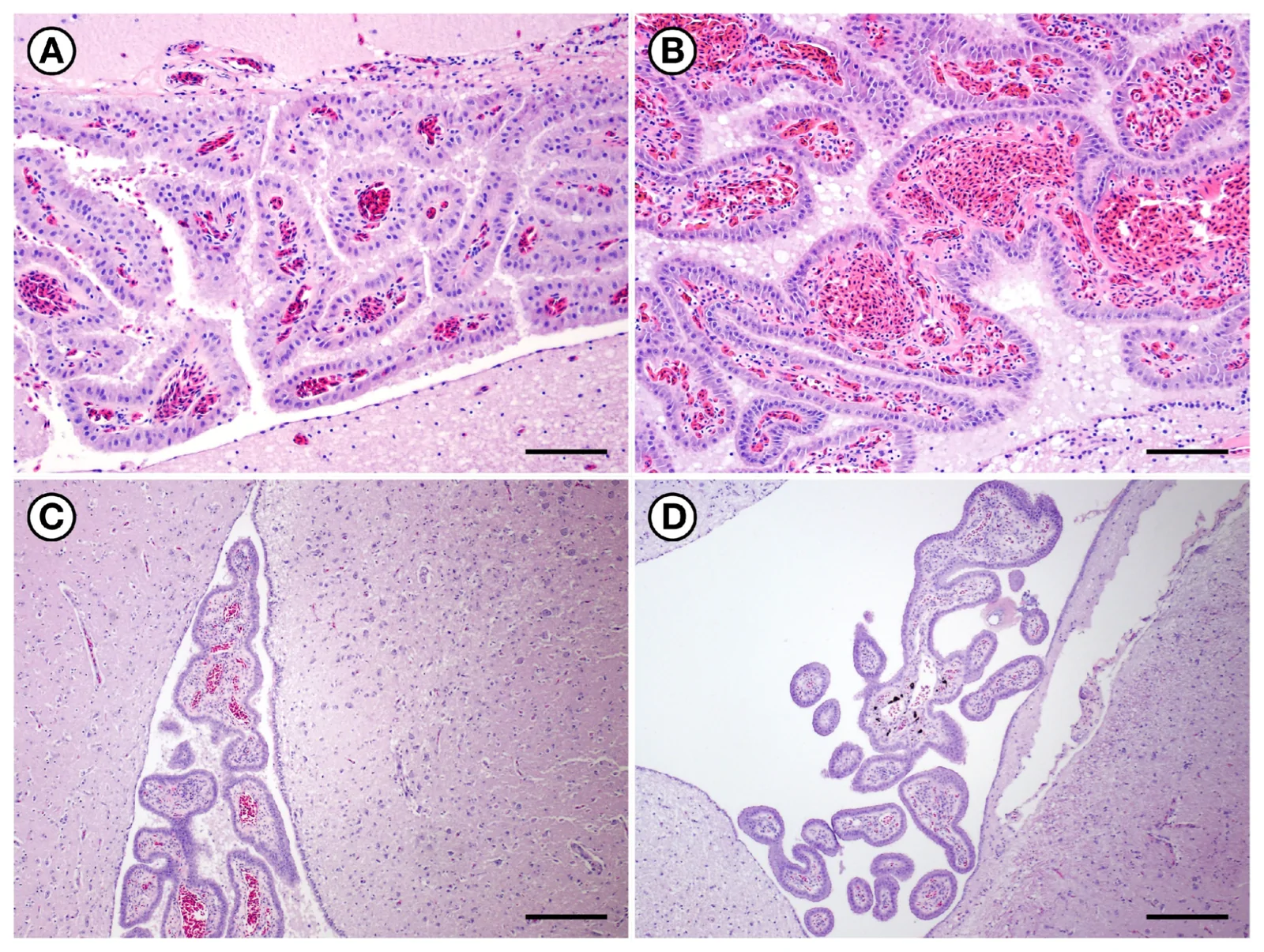
Choroid plexus pathology and ventricle dilation were common in brains of birds with acute and sublethal DA toxicosis. (**A**) Moderate diffuse stromal congestion of the choroid plexus, and mild ventricular microhemorrhage in an acute positive control BRPE (15-0100). (**B**) Markedly congested choroid plexus stroma from a PALO (17-0086) with acute, fatal DA toxicosis. (**C**) Choroid plexus and lateral ventricle from a PALO (IBR 17-0597) that stranded during the DA-associated mass-mortality event and was euthanized after four days of captive care. Choroidal congestion was within normal limits. Although not visible at this magnification, there was mild to moderate periventricular neuronal necrosis. (**D**) Choroid plexus and lateral ventricle from a PALO (17-0406) that stranded during the mass-mortality HAB event and was euthanized after 17 days of captive care. The choroid plexus appeared to be atrophic and hypoperfused, and the lateral ventricles were moderately dilated. All slides were stained with hematoxylin and eosin. Bars = 30, 30, 160, and 320 µm, respectively.

**Figure 9:**
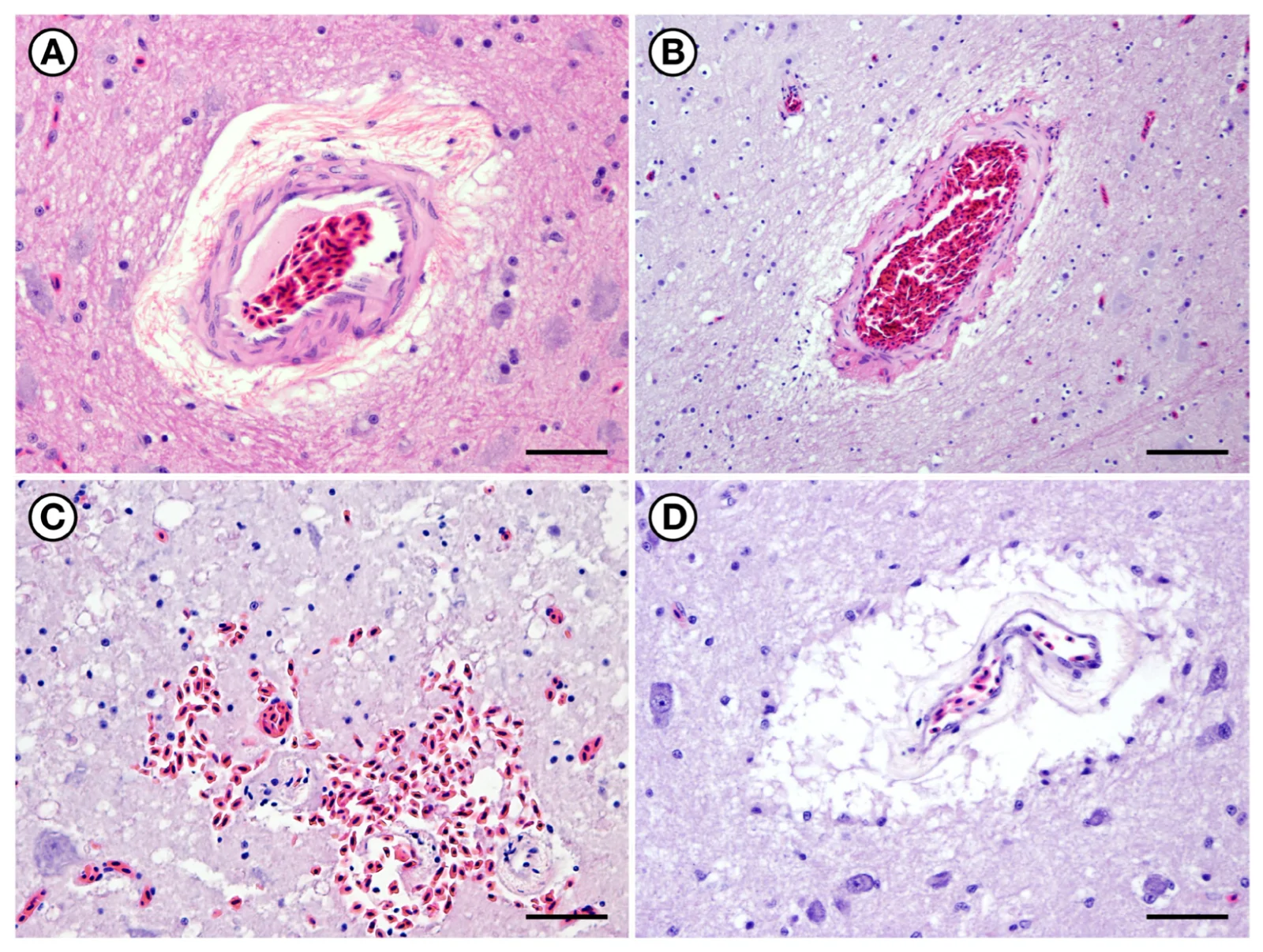
Vascular mural and perivascular pathology were common in brains of birds with lethal and sublethal DA toxicosis. (**A**) Normal cerebral arteriole from a negative control PALO (18-0503). (**B**) Mild perivascular spongiosis surrounding a cerebral arteriole from a positive control BRPE (15-0100). (**C**) Multiple small cerebral arteriolar profiles from a PALO (17-0086) with acute, fatal DA toxicosis. Decreased cytological detail of the arteriolar walls was associated with multifocal acute perivascular microhemorrhage and spongiosis. (**D**) Cerebral arteriole from a PALO (IBR 17-0406) that was euthanized after 17 days of care due to chronic neurological disease. Decreased cellularity and cytological detail in arteriolar walls was accompanied by enlargement of perivascular Virchow–Robin spaces (or the avian equivalent) and mild perivascular spongiosis. All slides were stained with hematoxylin and eosin. Bars = 60, 65, 30, and 30 µm, respectively.

**Figure 10:**
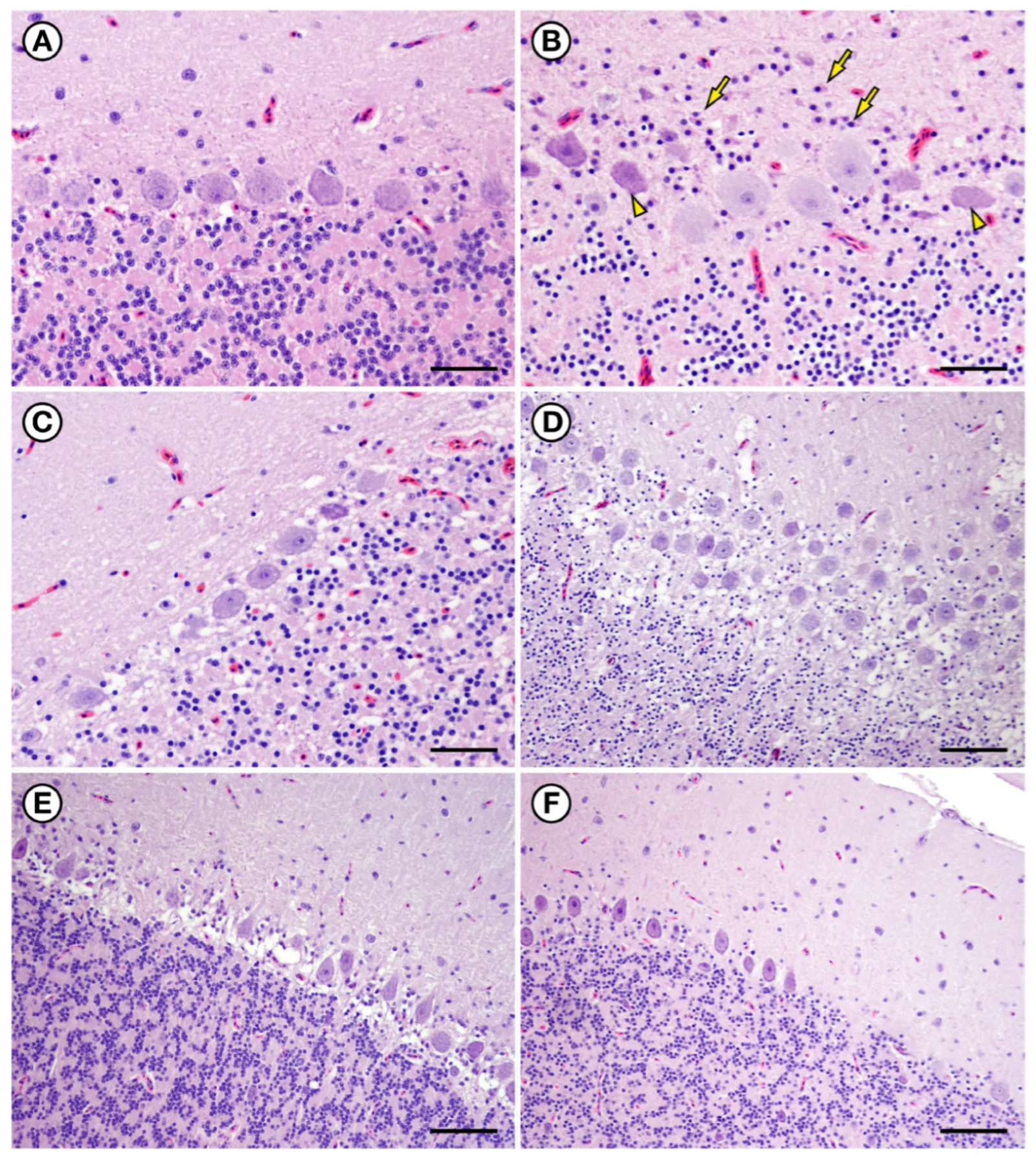
Pathology of the Purkinje cell layer was common in cerebellum of birds with acute fatal and sublethal DA toxicosis. (**A**) Normal cerebellar Purkinje cell layer from a negative control PALO (18-0503). Note the even distribution and appearance of the large Purkinje cell neurons and the absence of perineuronal spongiosis. (**B**) Cerebellar Purkinje cell layer from a positive control BRPE (15-0100). Some glial cells surrounding the Purkinje cell neurons (presumptive astroglia; arrows) exhibited homogenous eosinophilic to amphophilic cytoplasm suggestive of cell necrosis. Some Purkinje cells also appeared smaller than normal, with dense hyperchromic cytoplasm and possible nuclear karyolysis (arrowheads). (**C**) Cerebellar Purkinje cell layer from a PALO (17-0086) with acute, fatal DA toxicosis. Mild spongiosis was visible in tissue surrounding each Purkinje cell and some Purkinje cell neurons appeared smaller than normal, with dense hyperchromic cytoplasm and possible nuclear karyolysis. (**D**) Cerebellar Purkinje cell layer from a PALO (IBR 17-0597) that was euthanized four days post-stranding. Spongiosis surrounding each Purkinje cell neuron was very prominent and appeared to be due to cytoplasmic pallor and swelling of perineuronal glial swells. This section is tangential, resulting in mild artifactual lesion enhancement. (**E**,**F**) Cerebellar Purkinje cell layer from a PALO (IBR 17-0406) that was euthanized 17 days post-stranding. The cytoplasmic pallor and swelling surrounding each Purkinje cell were less prominent, but the arrangement of Purkinje cell neurons was irregular and uneven. All slides were stained with hematoxylin and eosin. Bars = 65, 65, 65, 160, 160, and 160 µm, respectively.

Three loons (two adult females and one adult male) with suspected acute fatal DA toxicosis were examined microscopically; results were summarized in Table S1. When compared to negative and positive control birds, these loons exhibited diffuse, moderate to marked brain congestion encompassing the neuropil, meninges, and ventricular choroid plexus (Figure 5, Figure 6, Figure 7 and Figure 8A,B). Edema and pinpoint hemorrhages (microhemorrhages) were visible in the meninges, perivascular neuropil, and brain ventricles, and were accompanied by mild perivascular, periventricular, and diffuse spongiosis (Figure 7C and Figure 9A–C). The cerebellar Purkinje cell layer exhibited possible neuronal degeneration and/or necrosis, gliosis, and mild laminar spongiosis with a pattern that suggested cytoplasmic swelling of perineuronal glial cells (Figure 10C).

**Figure 11:**
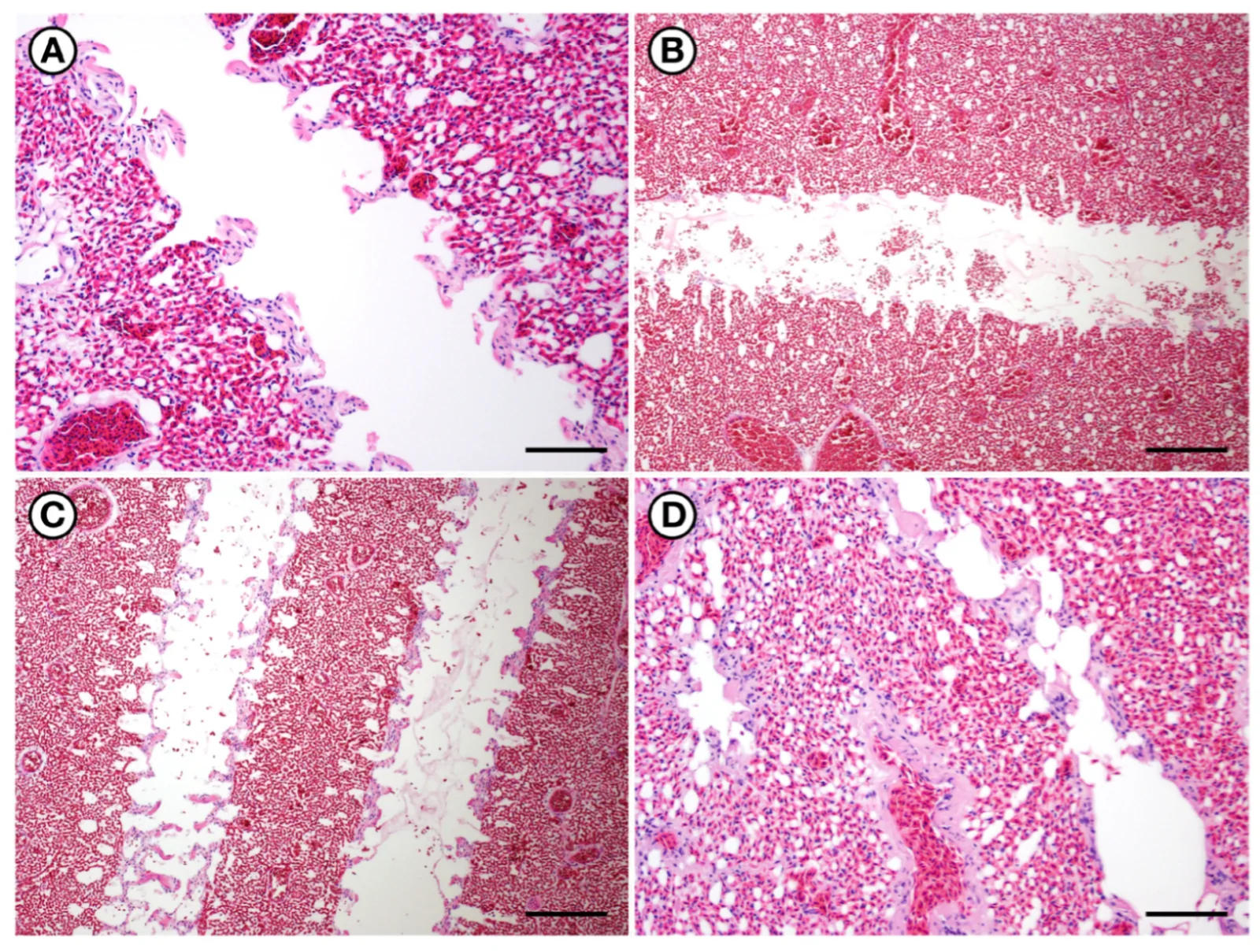
Mild to moderate, transient pulmonary pathology was common in birds with acute fatal DA toxicosis. (**A**) Normal lung and parabronchus from a negative control PALO (06-0219) for reference. (**B**) Lung and parabronchus from a positive control loon (07-0410); there was diffuse, severe vascular congestion, and the parabronchus was filled with proteinacious fluid and extravasated red blood cells. (**C**) Lung and parabronchus from a PALO (17-0086) with acute, fatal DA toxicosis; there was diffuse, severe vascular congestion, and the parabronchus was filled with proteinacious fluid and extravasated red blood cells. (**D**) Lung and parabronchus from a PALO (IBR 17-0406) that was euthanized 17 days post-stranding due to chronic neurological abnormalities. The pulmonary congestion, edema, and hemorrhage that were characteristic of birds that died acutely with high DA concentrations in GI content, tissues, and body fluids were no longer visible. All slides were stained with hematoxylin and eosin. Bars = 65, 160, 160, and 65 µm, respectively.

**Figure 12:**
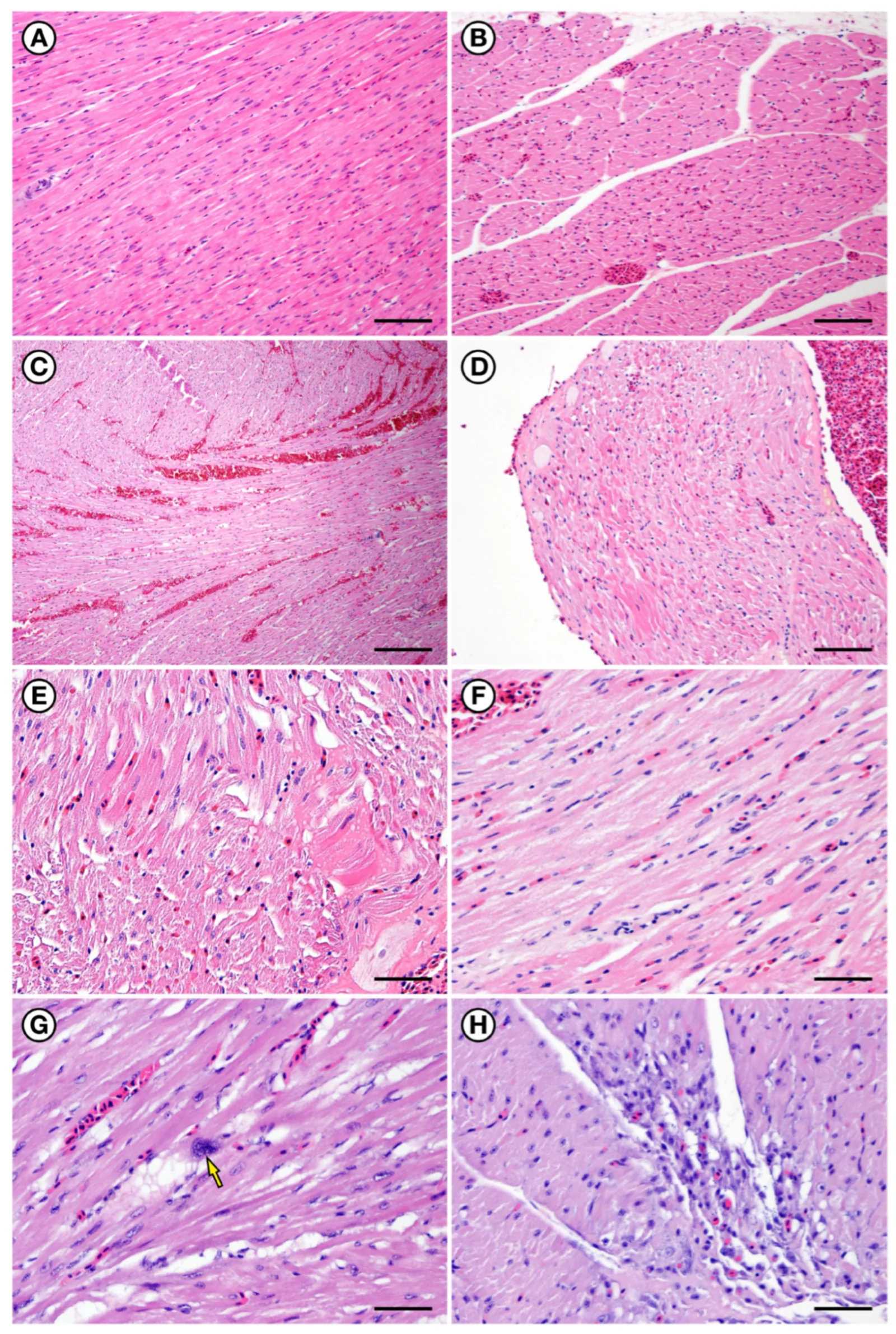
Cardiovascular pathology was common in birds with acute to chronic DA toxicosis. (**A**) Normal myocardium from a negative control PALO (18-0503). (**B**) Myocardium from a positive control BRPE (15-0100); there was moderate diffuse vascular congestion and possible mild interstitial edema. (**C**) Myocardium from a PALO (17-0086) with acute DA toxicosis; there was moderate diffuse vascular congestion and multifocal areas of blood pooling or microhemorrhage. (**D**) Myocardium from a PALO (17-0086) with acute DA toxicosis; although the vascular congestion was less severe, there was moderate variation in myofiber cytoplasmic staining. Some myofibers had pale, finely granular cytoplasm, while others exhibited homogenous, brightly eosinophilic cytoplasm suggestive of cell injury or necrosis. (**E**) Myocardium from a PALO (17-0086) with acute DA toxicosis; there was moderate variation in myofiber cytoplasmic staining and scattered bands of contracted, hypereosinophilic myofibers. (**F**) Myocardium from a PALO (IBR 17-0597) that was euthanized four days post-stranding; the variation in myofiber staining, with interspersed pale and hypereosinophilic myofibers, was especially prominent in this case. (**G**) Myocardium from a RTLO (IBR 17-0353) that was euthanized 18 days post-stranding; although the variation in myofiber staining was less prominent, the arrangement of myofibers was more haphazard, with intervening areas of pale, vacuolated adipocytes (presumptive fatty degeneration), and occasional enlarged, mis-shaped myofiber nuclei suggestive of abortive myofiber regeneration (arrow). (**H**) Focal area of myofiber loss, stromal collapse, and fatty degeneration in the myocardium of a RTLO (IBR 17-0353) that was euthanized 18 days post-stranding. All slides were stained with hematoxylin and eosin. Bars = 65, 65, 160, 65, 65, 65, 65, and 65 µm, respectively.

**Figure 13:**
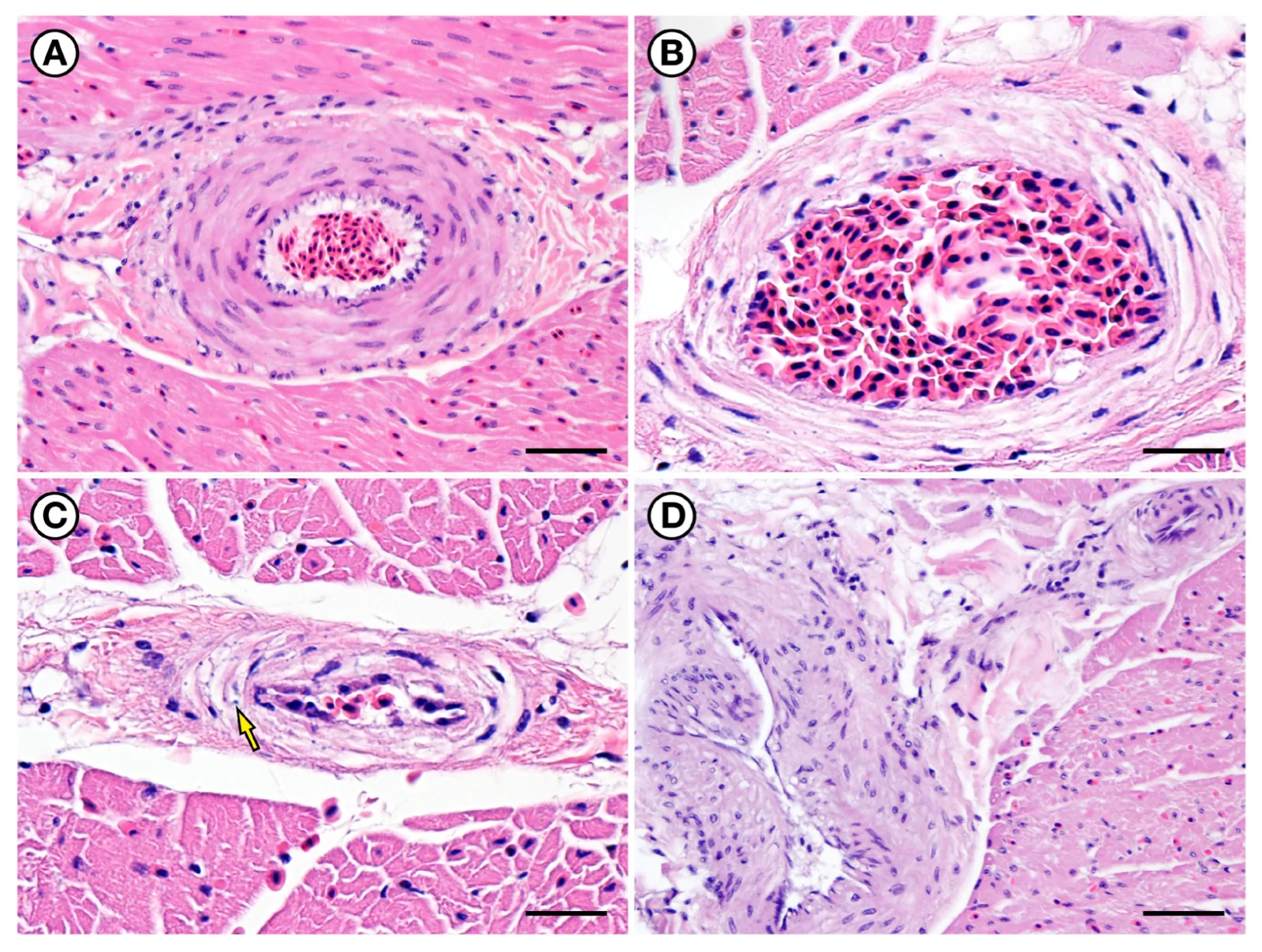
Mild to moderate coronary arterial pathology was common in birds with acute fatal and sublethal DA toxicosis. (**A**) Normal coronary arteriole from a negative control PALO (18-0503). (**B**) Coronary arteriole from a positive control BRPE (15-0100); mural smooth muscle cells were swollen, with pale cytoplasm and mild intercellular edema. (**C**) Coronary arteriole from a positive control BRPE (15-0100), showing suspected necrosis or apoptosis of mural smooth muscle cells, characterized by cell swelling, cytoplasmic pallor, and karyorrhectic nuclei (arrow). (**D**) Coronary arteriole from a PALO (IBR 17-0406) that was euthanized 17 days post-stranding; cell swelling and cytoplasmic pallor of mural smooth muscle cells were less severe than in the previous photos, but still apparent. All slides were stained with hematoxylin and eosin. Bars = 65, 30, 30, and 65 µm, respectively.

Microscopic lesions beyond the central nervous system in the loons suspected of acute DA toxicosis included diffuse, moderate to marked pulmonary vascular congestion, edema, and hemorrhage, which was similar to the findings in the positive control birds and in contrast with those in the negative control birds (Figure 11A,B). Diffuse congestion was also prominent in the liver, kidneys, adrenal glands, and heart (Figure 12A–D). Also noted were mild cardiac epicardial and perivascular microhemorrhages, edema, and patchy cytoplasmic pallor, hypereosinophilia, and vacuolation of cardiac myofibers suggesting myotoxicity and myodegeneration (Figure 12D,E). These findings were accompanied by mild cardiac arteriolar mural edema and pallor of mural smooth muscle cells (Figure 13A,B). Cardiac arteriolar mural edema was accompanied by necrosis or apoptosis of mural smooth muscle cells in loons with suspected acute DA toxicosis and the positive control birds (Figure 13C), but were not observed in the negative controls. All three loons in the acute DA toxicosis subcategory were diagnosed with fatal acute DA toxicosis based on findings from gross necropsy, toxicology, and histopathology. Observed pathology that could be secondary to the neurotoxic effects of DA included aspiration pneumonia, emaciation, trauma, and stranding-associated pododermatitis (Table S1).

### 2.3. Subacute to Chronic DA Toxicosis Suspects (4 to 86 Days Post-Stranding; n = 8)

Birds within the subacute to chronic DA toxicosis subgroup comprised live-stranded loons that spent between 4 and 86 days in rehabilitative care at IBR. Based on the number of days in care post-stranding and gross and microscopic lesion patterns, four birds were determined to have sublethal subacute DA toxicosis, and four had chronic sublethal DA toxicosis. Gross and microscopic findings and DA biochemical test results were summarized in Table S1. Because these birds spent time under veterinary observation, behavioral information and clinical history were also summarized.

#### 2.3.1. Subacute to Chronic DA Toxicosis Suspects Clinical History and Gross Necropsy (*n* = 8)

Eight loons with suspected subacute to chronic DA toxicosis, including birds with severe, chronic behavioral abnormalities were necropsied within 2 h of euthanasia, and samples were collected for biochemical testing and histopathology. Key clinical and gross necropsy findings and biochemical test results were summarized in Table S1. Birds with suspected subacute DA toxicosis had clinical signs such as dehydration and behavioral abnormalities, and gross necropsy findings included emaciation and congestion. Severe antemortem clinical abnormalities and/or grossly apparent brain pathology were observed in the four loons with chronic sublethal DA toxicosis that died or were euthanized 17 days or more post-stranding. Findings for this latter group of birds are summarized in greater detail below:

A PALO (17-0406) that was euthanized 17 days post-stranding exhibited chronic obtundation and somnolence, “floating oddly in the pool”, not diving, and lack of avoidance to capture (Supplementary Materials Video S1). These abnormalities were observed from hospitalization to the time of euthanasia. At necropsy this bird’s brain was found to be unusually diffusely soft and friable.

A RTLO (17-0353) was euthanized 18 days post-stranding due to progressive behavioral abnormalities: the bird was noted to be a “crazy diver” for the first 10 days of care but became progressively lethargic, obtunded, and somnolent over time, with an abnormal head and tail posture. Other antemortem findings included bilateral conjunctivitis and mild plumage problems including a few broken feathers. Humane euthanasia was performed at 18 days post-stranding when the bird became lethargic and was floating aimlessly. No gross abnormalities were observed at necropsy. 

**Figure 14:**
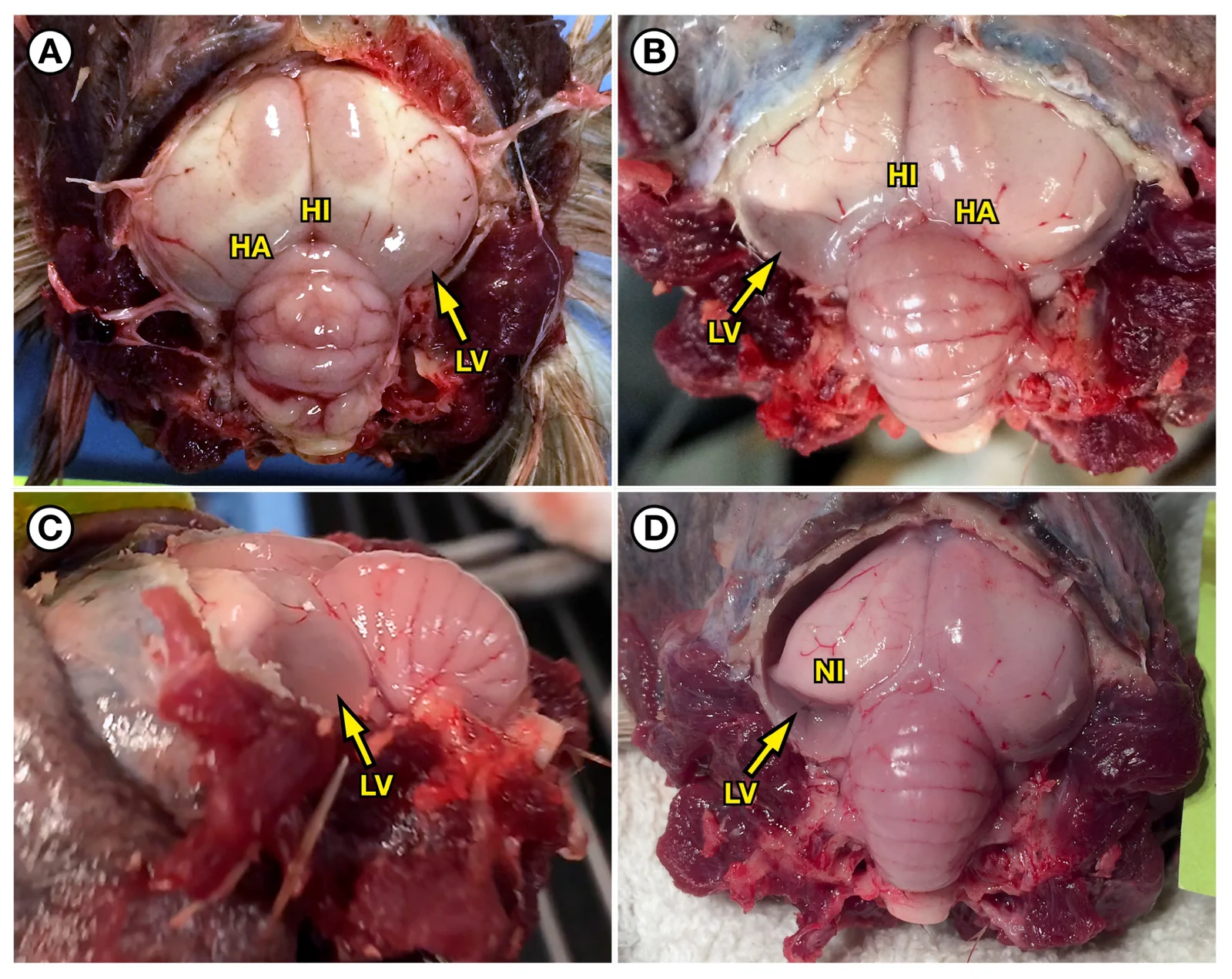
Birds surviving DA toxicosis for several weeks developed irreversible brain pathology that was especially severe and grossly apparent in the caudodorsal cerebrum. (**A**) Brain from a PALO (IBR 17-0316) that was euthanized 57 days post-stranding due to severe, chronic behavioral abnormalities. Gross necropsy revealed severe, diffuse pallor of the dorsal and caudal cerebrum, mild diffuse cerebellar enlargement, and suspected meningeal edema. In addition, the hippocampus (HI) and hyperpallium apicale (HA) were severely atrophic and transparent, revealing the underlying markedly dilated and fluid-distended lateral ventricles (LV). Brain tissue beneath the lateral ventricles (the dorsal nidopallium) was also abnormally pale and atrophied, contributing to the apparent ventricular enlargement. (**B**) Brain from a second PALO (IBR 17-0615) that was euthanized 86 days post-stranding with severe, chronic behavioral abnormalities. Gross findings in the brain were similar to those described in (**A**) above but were more asymmetrical. Note the severe tissue loss in the left caudodorsal cerebrum, resulting in marked enlargement of the left lateral ventricle. The cerebellum was also mildly and diffusely enlarged. (**C**) Left lateral view of the same brain (IBR 17-0615) shown in Figure 14B above, showing severe atrophy of brain tissue in the left caudodorsal cerebral hemisphere and marked enlargement and fluid distension of the left lateral ventricle (LV). (**D**) Caudodorsal view of the same brain (IBR 17-0615) shown in (**B**,**C**) after the hippocampus and hyperpallium apicale were removed over the left cerebral hemisphere, revealing the markedly enlarged lateral ventricle (LV) and the diffusely atrophic and pale dorsal nidopallium (NI) on the ventral surface of the lateral ventricle.

A PALO (17-0316) that was euthanized 57 days post-stranding due to avian poxviral dermatitis had a clinical history of episodic somnolence, obtundation, severe aggression, and colliding with inanimate objects. This bird was unusually easy to catch, not diving, and had chronic hypothermia. Necropsy revealed a thin bird with severe, bilaterally symmetrical pallor and swelling of the caudodorsal and dorsomedial cerebrum, and mild diffuse cerebellar swelling with markedly enlarged lateral ventricles that were distended with cerebrospinal fluid (CSF; Figure 14A). 

A PALO (17-0615) was euthanized 86 days post-stranding due to chronic somnolence and obtundation, absence of normal diving behavior, and being unusually easy to catch. These clinical abnormalities were first noted at 17 days post-stranding, with no improvement observed over the next 40 days of care. Although this bird was emaciated and dehydrated at the time of stranding, its nutritional condition had normalized while in care. Gross necropsy revealed marked thinning, atrophy, and increased translucency of the caudodorsal and dorsomedial cerebrum, and the lateral ventricles were severely enlarged and distended with CSF (Figure 14B,C). Although the lesions were bilateral, they were more severe in the left cerebral hemisphere (Supplementary Materials Video S2). The dorsal nidopallium, located immediately subjacent to the lateral ventricles, was also abnormally pale (Figure 14D). Possible mild diffuse cerebellar swelling was also observed.

#### 2.3.2. Subacute to Chronic DA Toxicosis Suspects Biochemical Testing (*n* = 8)

Postmortem samples from five of the eight loons (63%) in the suspected subacute to chronic DA toxicosis group that had been in care between 4 days and 86 days post-stranding were DA-positive in at least one collected tissue necropsy sample. DA was detected in 4/8 (50%) liver and kidney samples (Table S1). The five DA-positive loons had elevated DA concentrations detected in one or more of the postmortem tissue samples that were collected following 4 days to 57 days at a veterinary captive care facility where feed was confirmed to be DA-negative through biochemical screening by the supplier (McRobert’s Sales, Co., Inc., Ruskin, FL, USA). For these loons, DA was detected in 4/8 (50%) of liver and kidney samples (Table S1). Interestingly, one loon with severe chronic behavioral abnormalities and severe, grossly apparent brain lesions was DA-positive in liver, kidney, and spleen tissues after spending 57 days in captive care, with DA concentrations as high as 230 ng g^−1^ (Table S1). 

#### 2.3.3. Histopathology for Subacute DA Toxicosis Suspects (4 to 15 Days Post-Stranding; *n* = 2)

**Figure 15:**
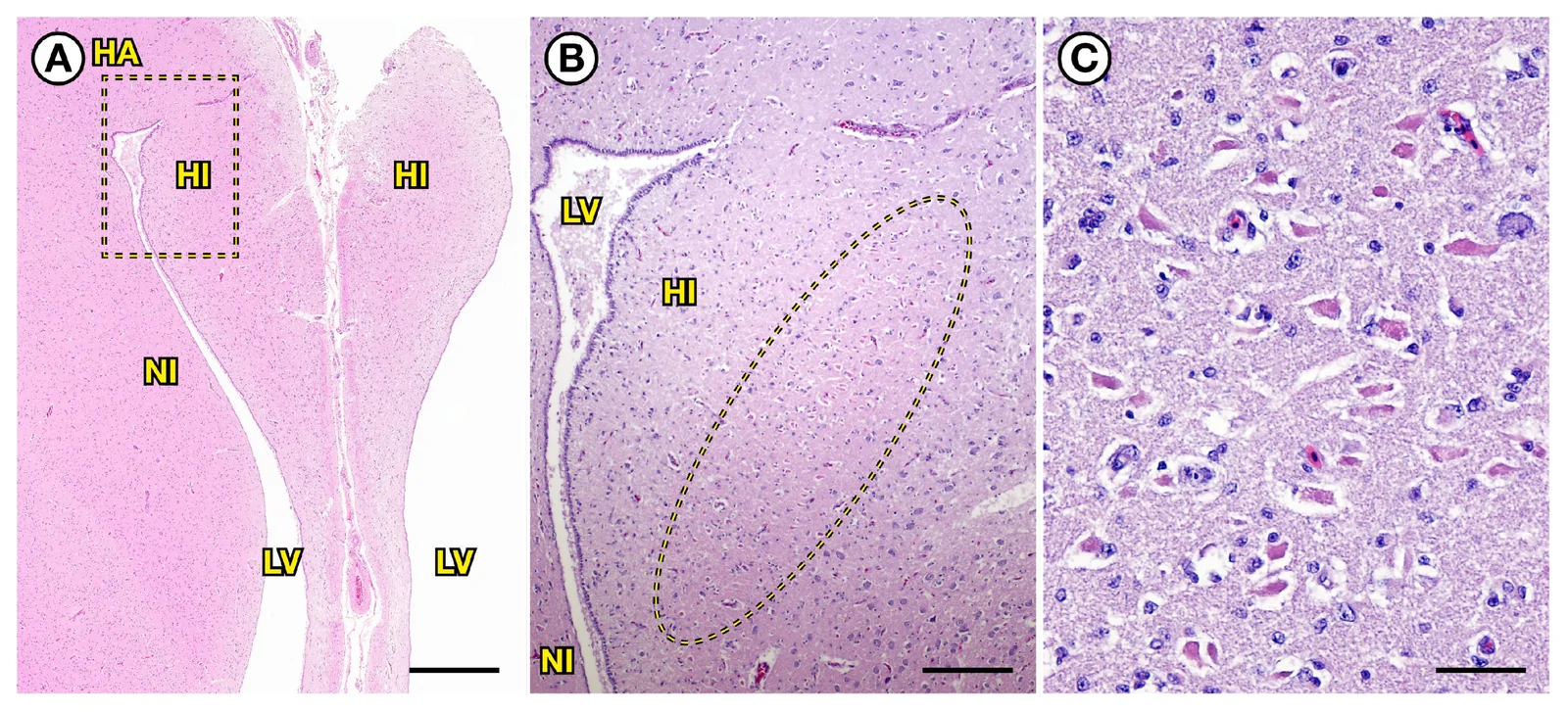
Severe neuronal necrosis was visible within the cerebral hippocampus of a loon that died due to subacute DA toxicosis. (**A**) Low-magnification view of caudal cerebrum from a PALO (IBR 17-0597) that was euthanized 4 days post-stranding. The severe congestion of the cerebrum, meninges, and choroid plexus that characterized brains of loons that were recovered freshly dead during the mass-mortality event was no longer visible. Locations of the hippocampus (HI), hyperpallium apicale (HA), dorsal nidopallium (NI), and lateral ventricles (LV) are shown. Tissue within the dashed line box is shown at higher magnification in (**B**). Lateral tissue displacement on the right side of the section is artifactual. (**B**) Higher-magnification view of the caudodorsal cerebral midline of the same PALO (IBR 17-0597) as in (**A**). Locations of the hippocampus (HI), dorsal nidopallium (NI), and lateral ventricles (LV) are shown. A linear band of swollen neurons with brightly eosinophilic cytoplasm was visible in the hippocampus (inside of the ellipse), and scant proteinaceous material was present in the ventricle (LV). (**C**) (IBR 17-0597) Higher-magnification view of the area of hippocampus delineated by the ellipse in (**B**) above, showing the linear band of swollen neurons with brightly eosinophilic cytoplasm (neuronal necrosis) accompanied by mild to moderate perineuronal gliosis. All slides were stained with hematoxylin and eosin. Bars = 650, 160, and 30 µm, respectively.

**Figure 16:**
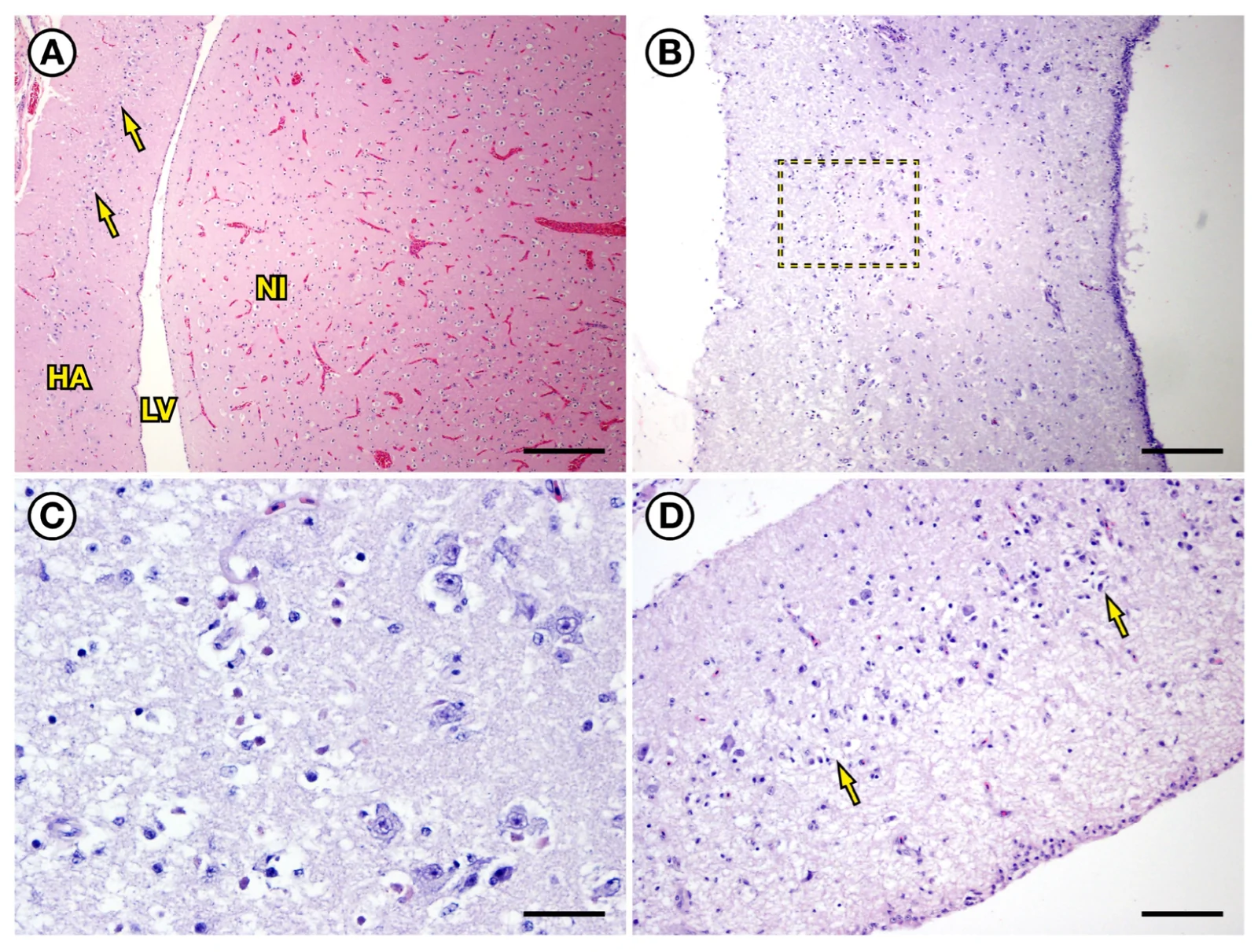
Severe neuronal necrosis was visible in the hyperpallium apicale region of the caudodorsal cerebrum of a loon that died due to subacute DA toxicosis. (**A**) Hyperpallium apicale (HA), lateral ventricle (LV), and dorsal nidopallium (NI) from the caudodorsal cerebrum of a PALO with acute, fatal DA toxicosis (17-0086), shown for reference. Although there was marked diffuse congestion, the tissue was otherwise microscopically unremarkable. Even at this low magnification, a prominent linear band of neurons is visible running longitudinally along the HA (arrows). (**B**) Mild to moderate diffuse tissue pallor, spongiosis, and gliosis in the cerebral HA from a PALO (IBR 17-0597) that was euthanized 4 days post-stranding. The region within the dashed box is shown at higher magnification in (**C**). (**C**) (IBR 17-0597) Higher-magnification inset from (**B**) above; numerous small neurons and/or astroglia are necrotic, characterized by brightly eosinophilic cytoplasm, karyorrhexis, and karyolysis. (**D**) HA from a PALO (IBR 17-0406) that was euthanized after 17 days in care. There was diffuse tissue pallor, spongiosis, and gliosis, and the linear band of neurons running longitudinally along the HA was partially disrupted due to neuronal loss(arrows; compare with (**A**)). All slides were stained with hematoxylin and eosin. Bars = 325, 160, 30, and 60 µm, respectively.

**Figure 17:**
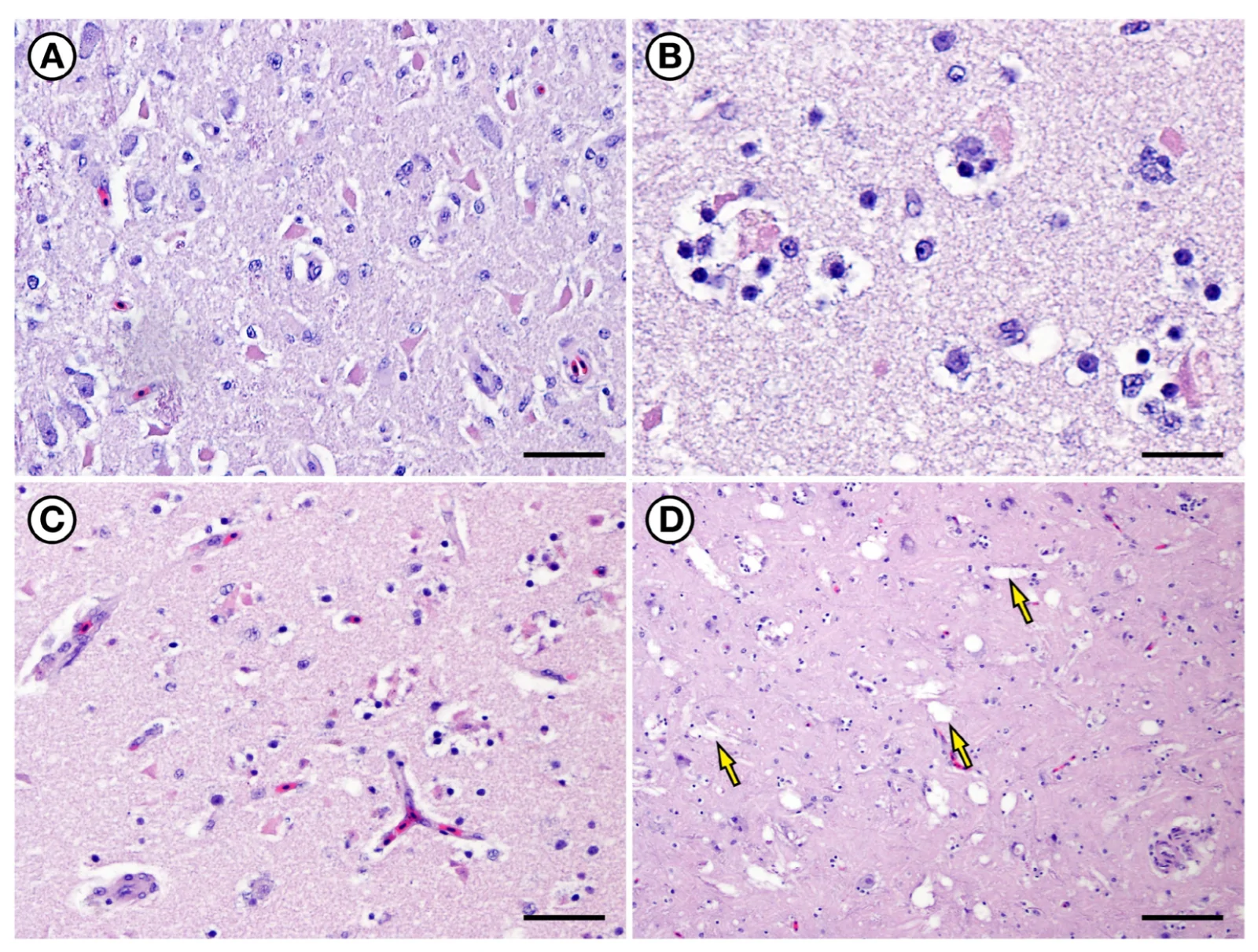
Severe neuronal necrosis, neurophagocytosis, and astrogliosis were visible in the cerebrum of a loon that was euthanized due to subacute DA toxicosis. (**A**) Numerous swollen, hypereosinophilic (aka “red dead”) neurons and moderate gliosis in the hippocampus of a PALO (IBR 17-0597) that was euthanized 4 days post-stranding. (**B**) (IBR 17-0597) In some areas of the brain, swollen glial or phagocytic cells with prominent pale cytoplasm surrounded and were phagocytosing necrotic neurons (neurophagocytosis). (**C**) (IBR 17-0597) Areas of neuronal necrosis and neurophagocytosis were often accompanied by perilesional vascular hypertrophy. (**D**) (IBR 17-0597) Multifocal axonal degeneration (arrows) was due to necrosis of the corresponding neurons. All slides were stained with hematoxylin and eosin. Bars = 35, 16, 35, and 65 µm, respectively.

Histopathology of the cerebrum from a PALO (17-0597) that was euthanized 4 days post-stranding (Table S1) exhibited acute severe neuronal and glial necrosis characterized by mild to moderate cytoplasmic swelling or shrinkage, homogenous brightly eosinophilic cytoplasm, and karyolysis (Figure 15). Although multiple areas of the cerebrum were affected, the most extensive lesions were observed in the hippocampus, hyperpallium apicale, and dorsal nidopallium, the same anatomic areas that exhibited some of the most severe congestion in suspected acute DA toxicosis loons (Figure 16A). At all timepoints following DA exposure, the most severely affected areas of the brain were in these same areas of the caudodorsal and dorsomedial cerebrum. In the subacute DA toxicosis loons a prominent row of necrotic neurons was visible within the hippocampus (Figure 15C), and a linear band of smaller, acutely necrotic neurons or glial cells was visible in the hyperpallium apicale (Figure 16B,C). A more random pattern of neuronal and glial necrosis was present in the dorsal nidopallium. Areas of cell necrosis were characterized by mild vascular congestion, moderate to severe perivascular to diffuse spongiosis, moderate patchy gliosis, and mild axonal degeneration. More severely affected areas had prominent neuronal fragmentation, axonal degeneration, and glial satellitosis with many perineuronal glial cells exhibiting pale, swollen, vacuolated cytoplasm (Figure 17). 

**Figure 18:**
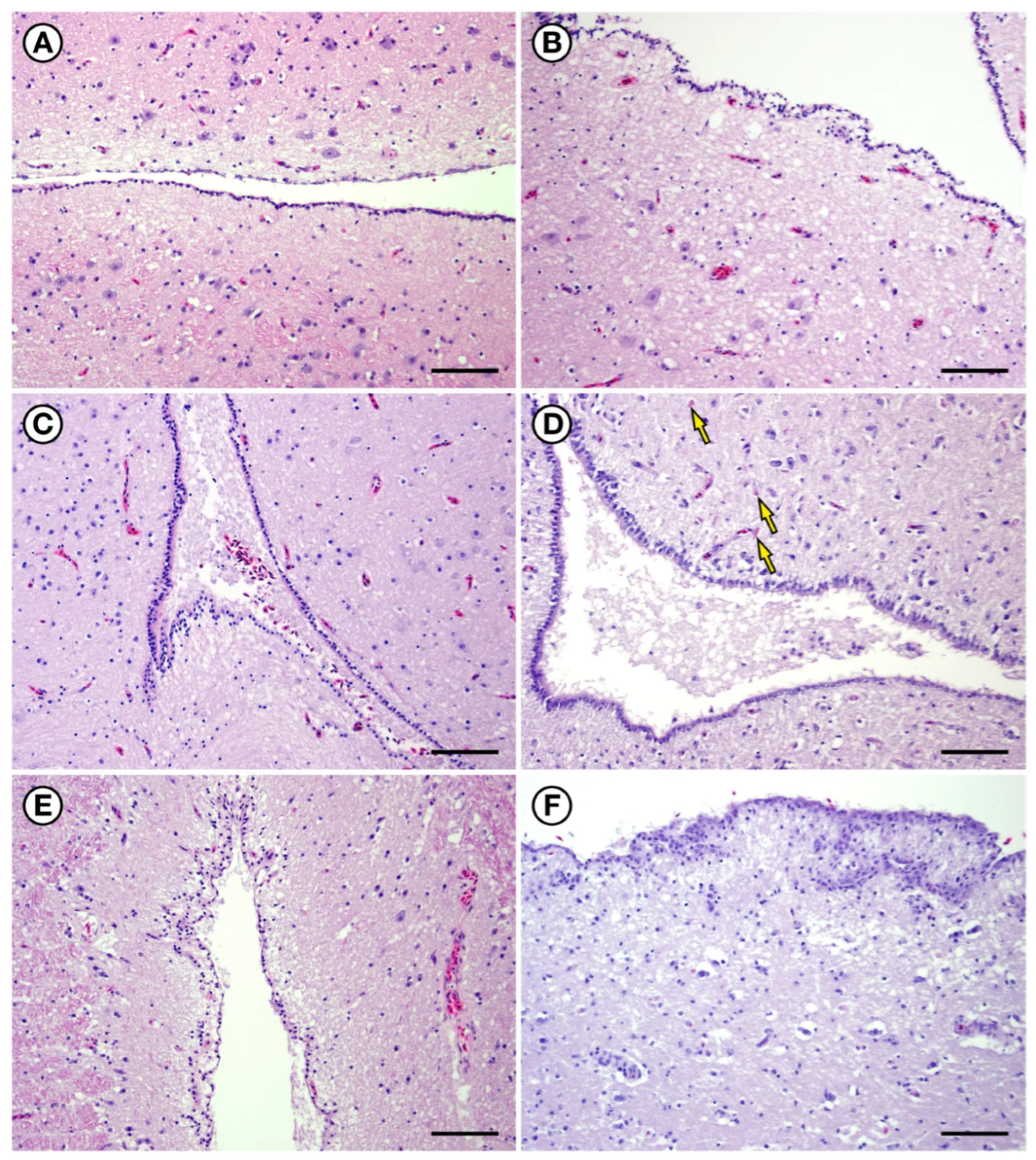
Ventricular and periventricular pathology was common in the cerebrum of birds with DA toxicosis. (**A**) Microscopic view of normal lateral ventricle from a negative control PALO (18-0503). (**B**) Lateral ventricle from a positive control BRPE (15-0100); the ependymal surface was irregular, with mild cytoplasmic vacuolation of ependymal cells, and the periventricular neuropil was congested and spongiotic. (**C**) Lateral ventricle from a PALO (17-0086) with acute, fatal DA toxicosis; the periventricular neuropil was congested and spongiotic, and there was scant hemorrhage admixed with proteinacious material in the ventricular lumen. (**D**) Lateral ventricle from a PALO (IBR 17-0597) that was euthanized 4 days post-stranding; the periventricular neuropil was congested and spongiotic, the ventricular lumen contained proteinacious material, and several necrotic (e.g., “red dead”) neurons were visible near the ventricles (arrows). (**E**) Lateral ventricle from a RTLO (IBR 17-0353); that was euthanized 18 days post-stranding. Although the vascular congestion had normalized, there was moderate periventricular spongiosis, and the ventricular surface was irregular and uneven with a partial double layer of ependymal cells. (**F**) Lateral ventricle from a PALO (IBR 17-0406) that was euthanized 17 days post-stranding; There was moderate perivascular spongiosis, and the ventricular surface was irregular and uneven, with multiple layers of ependymal cells forming small clefts and invaginations. All slides were stained with hematoxylin and eosin. Bars = 160, 65, 160, 160, 160, and 160 µm, respectively.

When compared to negative control birds (Figure 18A), early ventricular and periventricular lesions in positive control, suspected acute, and early subacute DA toxicosis loons exhibited possible mild ventricular dilation, meningeal edema, and periventricular spongiosis that was especially prominent around the lateral ventricles. Some ependymal cells were mildly swollen with pale, vacuolated cytoplasm, while the lumen of the lateral ventricles contained scant exfoliated ependymal cells, cell debris admixed with proteinaceous material, and mild hemorrhage (Figure 18B–D). The cerebellar Purkinje cell layer was characterized by moderate cytoplasmic swelling and pallor of peri-neuronal glial cells (Figure 10D). Mild to moderate edema was visible in larger white matter tracts throughout the brain, especially in the optic tectum, cerebellum, and adjacent tissues.

Cardiac histopathology revealed mild diffuse congestion, epicardial hemorrhage, and hyalinization of coronary arterioles with sparse mural mineralization. Mild to moderate variation in myofiber staining was associated with mild rhabdomyolysis (Figure 12F). Mild diffuse pulmonary congestion, parabronchial hemorrhage and edema and scattered heterophilic foreign body granulomas suggestive of aspiration pneumonia were also noted. Mild to moderate diffuse congestion was also observed in the liver, kidneys, and adrenal glands. 

The cerebrum from a RTLO (17-0439) that was euthanized 15 days post-stranding (Table S1) exhibited mild diffuse congestion, mild meningeal and ventricular edema and microhemorrhage, and mild perivascular to diffuse spongiosis. Patchy periventricular glial cell swelling and spongiosis were associated with mild ventricular dilation. 

Cardiac histopathology revealed myocardial congestion that was associated with foci of mild epicardial microhemorrhage, admixed pale and hypereosinophilic myofibers (presumed myonecrosis), and decreased smooth muscle density plus minimal mural hyalinization in coronary arterioles (Figure 13D). Mild to moderate pulmonary congestion was associated with moderate parabronchial hemorrhage and edema. There was also diffuse mild to moderate hepatic and renal congestion. 

#### 2.3.4. Histopathology for Chronic DA Toxicosis Suspects (17 to 86 Days Post-Stranding; *n* = 3)

Four loons that were euthanized between 17 days and 86 days post-stranding had an antemortem history of severe chronic neurological abnormalities, and three had grossly apparent encephalomalacia (Table S1). Unfortunately, formalin-fixed tissues were misplaced for one bird (17-0316), and tissues from a second loon (17-0615) were accidentally preserved in a fluid other than formalin (possibly isopropanol), thereby limiting histological assessment. Formalin-fixed tissues were examined from three of the four birds, including the loon with previously mentioned fixation artifact. 

**Figure 19:**
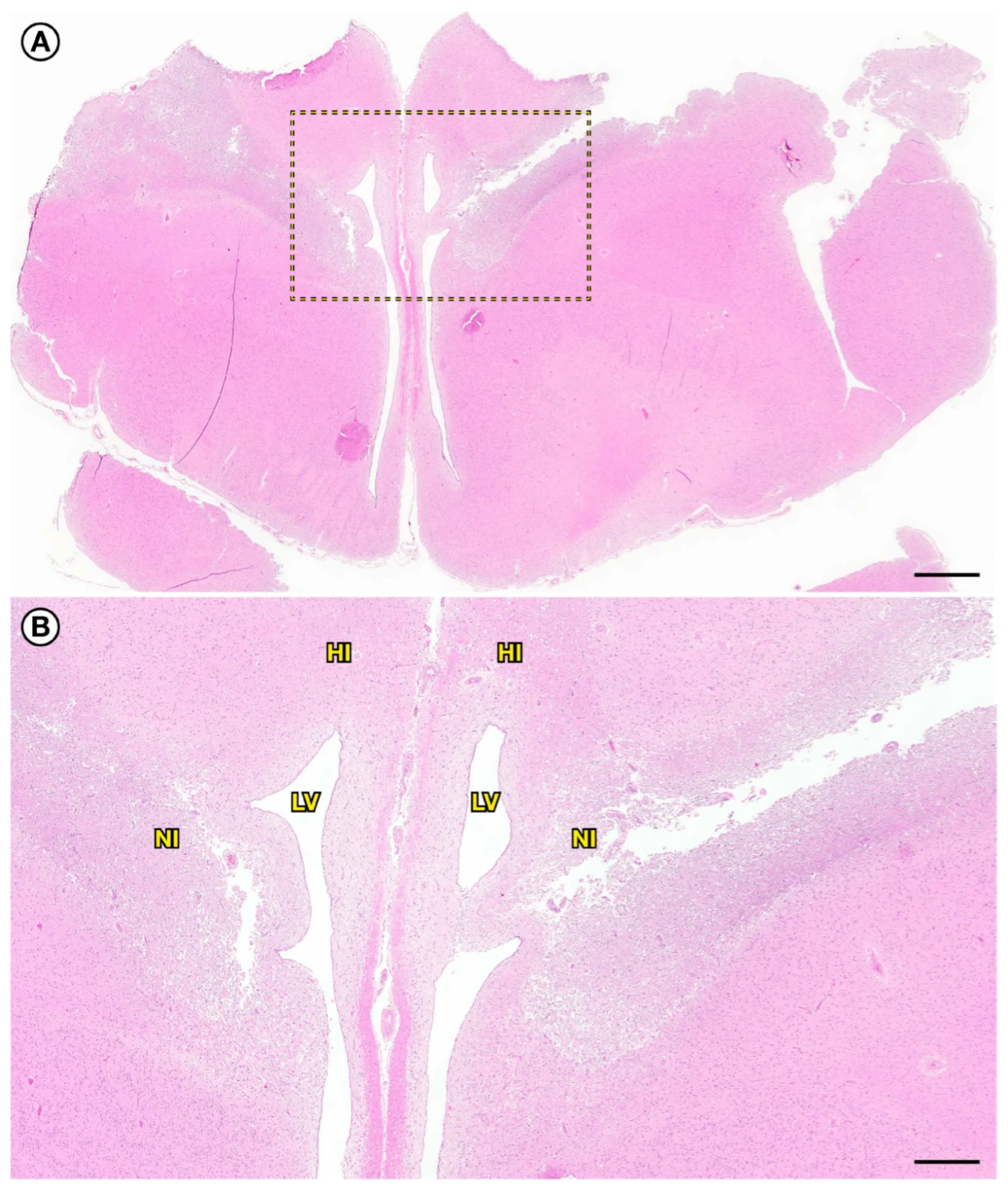
Chronic sublethal DA toxicosis in loons was associated with severe, variably symmetrical degeneration and atrophy of the caudodorsal periventricular cerebrum. (**A**) Low-magnification view of caudal cerebrum from a PALO (IBR 17-0406) that was euthanized 17 days post-stranding. Severe, well-demarcated, bilaterally symmetrical encephalomalacia and tissue atrophy were visible in the caudodorsal cerebrum. This lesion was most severe along the periventricular midline, and extended laterally as a V-shaped band of malacic cerebral tissue that surrounded the moderately dilated lateral ventricles. (**B**) (IBR 17-0406) Higher-magnification view of the region of the dorsal cerebral midline delineated by a box in the previous photo. Well-defined areas of grossly apparent, bilaterally symmetrical encephalomalacia and tissue atrophy in the dorsal, dorsolateral, and dorsomedial cerebrum corresponded with the locations of the hippocampus (HI), dorsal nidopallium (NI), and lateral ventricles (LV) (compare with the negative control tissue in Figure 5). All slides were stained with hematoxylin and eosin, with images obtained using a digital scanning microscope. Bars = 1500 and 500 µm, respectively.

**Figure 20:**
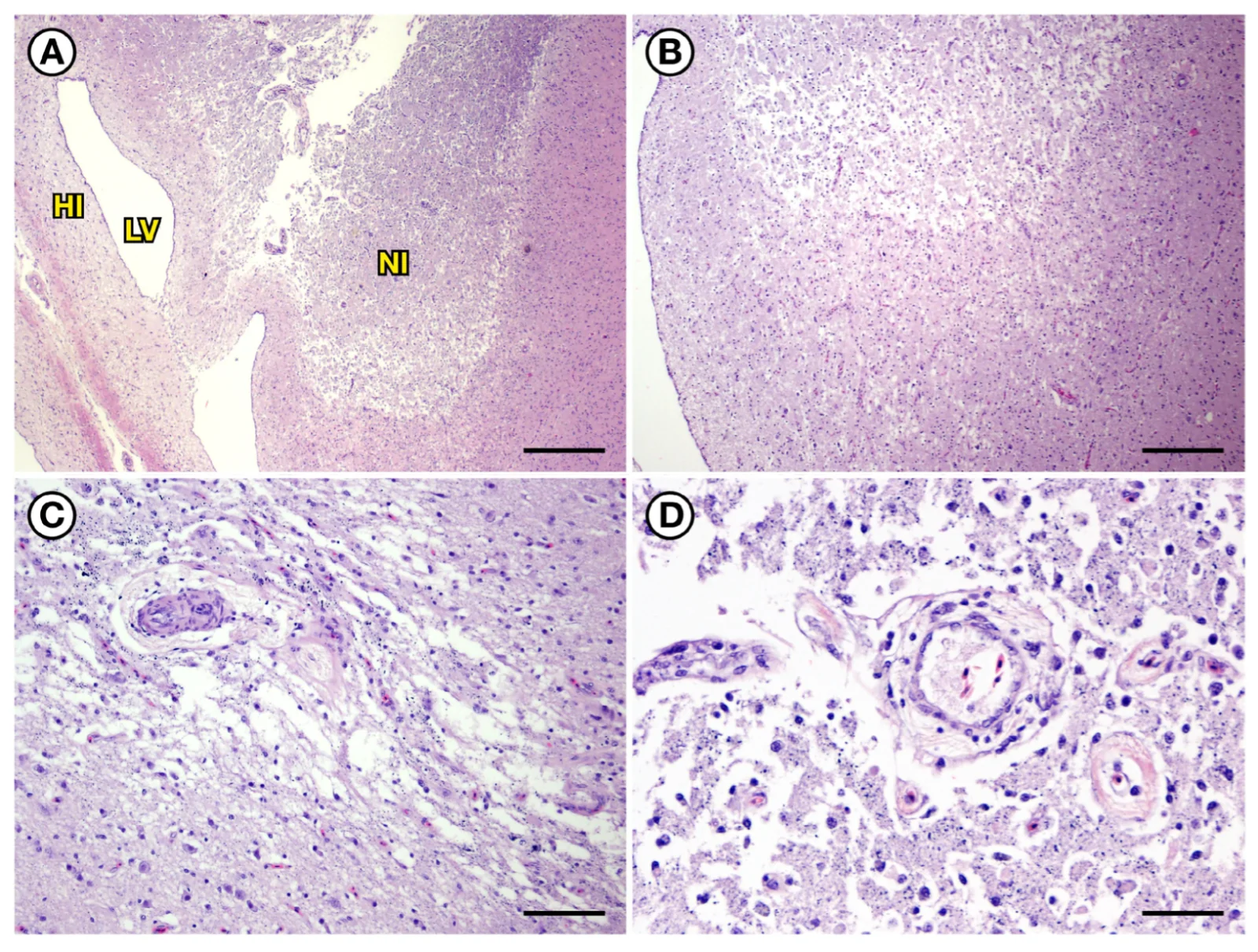
Severe atrophy, malacia, and dystrophic mineralization of the caudodorsal cerebrum were visible in loons with chronic neurological deficits due to DA toxicosis that survived ≥17 days post-stranding. (**A**) Caudodorsal cerebrum of a PALO (IBR 17-0406) that was euthanized 17 days post-stranding. Grossly, the caudodorsal cerebrum was soft, opaque, pale, and edematous. On the microscope, a large, well-demarcated region of tissue atrophy, malacia, and partial cavitation encompassed much of the dorsal nidopallium (NI) and the hippocampus (HI), and there was moderate dilation of the lateral ventricle (LV). (**B**) (IBR 17-0406) Higher-magnification view of the tissue shown in (**A**); the central zone of cerebral malacia and cavitation was surrounded by gliotic and spongiotic neuropil with prominent vascular hypertrophy. (**C**) (IBR 17-0406) Tissue malacia was associated with accumulation of glial cells, cell debris, and grey to black granular pigment. (**D**) (IBR 17-0406) In severely affected areas, normal brain tissue was replaced by cavitated tissue filled with glial cells, cellular debris, grey to black granular pigment, and dystrophic mineralization. All slides were stained with hematoxylin and eosin. Bars = 325, 160, 65, and 40 µm, respectively.

Microscopic examination of an abnormally soft brain from a PALO (17-0406) that was euthanized 17 days post-stranding revealed broad, bilaterally symmetrical bands of severely malacic neuropil that extended across the caudodorsal and dorsomedial cerebral hemispheres from the median longitudinal fissure to the dorsolateral cerebral surfaces (Figure 19 and Figure 20A,B). These lesions were composed of a central, well-demarcated zone of malacia and partial tissue cavitation surrounded by an outer rim of hypercellular, gliotic neuropil (Figure 20A,B). Within the severely malacic regions, reduced neuronal density and mild axonal degeneration were associated with accumulation of grey to black granular pigment and partially mineralized cell debris (Figure 20C,D). Variable vascular hypertrophy and moderate to marked perivascular to diffuse spongiosis and edema were also present, although without the severe vascular congestion that was typical of acute cases (Figure 7D). In some areas, the pattern of edema was suggestive of marked expansion of perivascular Virchow–Robin spaces (or the avian equivalent) with acellular, low-protein fluid (Figure 9D). 

The most severely affected regions of the cerebrum encompassed the hippocampus, hyperpallium apicale, dorsal nidopallium, and lateral ventricles. The lateral ventricles were moderately dilated with patchy swelling and pallor of the ventricular ependyma, while the choroid plexus appeared hypoperfused and was mildly atrophic in some areas (Figure 8D). There was mild to moderate periventricular spongiosis, gliosis, neuronal degeneration, and swelling and cytoplasmic pallor of glial cells around the lateral ventricles (Figure 16D). The ventricle surface was folded and irregular with abortive attempts at ependymal regeneration, which, in some areas, was characterized by multiple layers of ependymal cells, scattered regions of missing ependyma, and entrapment of small clusters of ependyma deep below the ventricle surface (Figure 18E,F). 

Moderate perivascular to diffuse spongiosis and gliosis without tissue cavitation were also visible in other areas of the cerebrum, cerebellum, and brainstem. Mild to moderate edema was visible in some of the larger white matter tracts, especially in the optic tectum, cerebellum, and nearby tissues. Moderate diffuse meningeal edema was accompanied by minimal multifocal meningeal and ventricular microhemorrhage. Patchy, minimal to mild swelling, cytoplasmic pallor, and a variation in staining of perineuronal glial cells were visible in the cerebellar Purkinje cell layer, with more chronic cases exhibiting patchy areas of reduced Purkinje neuron density (Figure 10E,F). 

The pulmonary congestion and edema that were characteristic of loons with acute DA toxicosis were no longer visible in birds that died at 17 or more days post-stranding (Figure 11D). Myocardial histopathology revealed patchy (mild to marked) variation in myofiber staining, from pale eosinophilic with granular-appearing and occasionally vacuolated cytoplasm, and small contracted myofibers with dark pink cytoplasm (Figure 12G). Anitchkow-type myofiber nuclei were relatively common along with moderate patchy anisokaryosis and occasional large, misshapen nuclei suggestive of abortive attempts at myofiber regeneration (Figure 12G) and patchy mild multifocal myofiber atrophy. In more chronic DA toxicosis suspects, scattered areas of haphazard cardiac myofiber arrangement with intervening adipocytes were suggestive of early fatty replacement, and occasional small foci exhibited near-total myofiber loss, scattered myofiber fragments, and fatty replacement (Figure 12H). A few small arterioles exhibited mild to moderate mural hyalinization and marked expansion of perivascular spaces. Mild epicardial edema and minimal epicardial hemorrhage were also present. 

Brain histopathology for the PALO (17-0353) that was euthanized at 18 days post-stranding (Table S1) yielded findings similar to those of the previous bird, PALO (17-0406), but without the striking bilaterally symmetrical encephalomalacia. Observed lesions included multifocal brain and meningeal edema, mild cytoplasmic swelling, pallor of the periventricular glial cells, and periventricular spongiosis. Mild to moderate dilation of the lateral ventricles was accompanied by scant ventricular microhemorrhage and ependymal vacuolation, regeneration, and scarring.

Myocardial histopathology revealed moderate to marked myonecrosis with fatty replacement, mild epicardial microhemorrhage, and coronary arteriolar hyalinization. Lesions were most severe in the ventricles, with the right ventricle containing a focal subendocardial area of near-total myofiber loss as well as sparse remaining myofiber fragments and fatty replacement. Moderate pulmonary hemorrhage and edema, and mild pectoral muscle rhabdomyolysis and fibrosis were also observed. 

The anatomic distribution of the severe, grossly apparent cerebral atrophy and ventricular dilation for the two loons (17-0316, 17-0615) that were euthanized in part due to chronic severe neurological disease at 57 (Figure 14A; 17-0316) and 86 days (Figure 14B–D; 17-0615) post-stranding (Table S1) corresponded spatially with the regions of severe, bilaterally symmetrical encephalomalacia in the loon that was euthanized at 17 days post-stranding (Figure 19 and Figure 20A,B; 17-0406). Unfortunately, formalin-fixed tissues were misplaced for the loon euthanized at 57 days (17-0316), and tissues collected during necropsy from the other bird (17-0615) were inadvertently fixed in isopropanol or some other coagulative fixative, thereby limiting histological interpretation. However, marked dilation of the lateral ventricles and severe atrophy, rarefaction, and cavitation of the dorsomedial and caudodorsal periventricular cerebral neuropil were confirmed microscopically for the latter case (17-0615), and cardiac pathology included mild myocardial hemorrhage, edema, myonecrosis, and myofiber atrophy. Mild diffuse pulmonary congestion, hemorrhage, and edema were also noted.

### 2.4. Positive Controls for Acute DA Toxicosis (n = 2)

Two birds found dead during DA-associated events that were temporally and spatially unrelated to the 2017 DA-associated stranding event investigated here were included in the study as positive controls: a subadult male brown pelican (*Pelecanus occidentalis*; BRPE) and an adult female RTLO.

The subadult male BRPE (Table S1; 15-0100) was found dead in Monterey County, California in 2015 during a biochemically confirmed DA event with localized mass-mortality of northern anchovies (*Engraulis mordax*), a common prey item for BRPE. A pooled sample of three whole anchovies from this event contained very high concentrations of DA (170,000 ng g^−1^). The pelican carcass was found near dead anchovies and partially digested small fish fragments, and anchovy otoliths were recovered from its GI tract. Postmortem cloacal content, digesta, and pericardial fluid from the pelican contained up to 285,000 ng g^−1^ DA (Table S1).

At necropsy, the pelican was in good nutritional condition, suggestive of rapid death. Diffuse, moderate to marked vascular congestion and focal venodilation were visible in the brain (Figure 2B), and abundant clear, red-tinged fluid and white froth were present in the lungs and air sacs. There was diffuse congestion of all major tissues, and the systemic veins and heart were acutely dilated and blood-filled, accompanied by moderate serous pericardial effusion (Figure 3). 

Histopathology revealed moderate to marked diffuse congestion that was especially prominent in the brain (Figure 2B), lungs, heart (Figure 3), liver, adrenal glands, and kidneys. There was marked choroidal congestion in the brain (Figure 8A), along with mild patchy ventricular and peri-choroidal hemorrhage, white matter rarefaction, and perivascular (Figure 9B) and periventricular (Figure 18B) spongiosis. Scattered glial cells in the cerebellar Purkinje cell layer appeared mildly shrunken, with hyper-eosinophilic cytoplasm (Figure 10B). Minimal heterophilic and nonsuppurative perivascular cuffing was observed in a few cerebral and meningeal venules and capillaries. 

Microscopic examination of the heart revealed mild multifocal epicardial and myocardial microhemorrhage along with perivascular edema. Marked diffuse vascular congestion was most apparent in the superficial myocardium and epicardial adipose (Figure 12B). Variable staining of cardiac myofibers was especially prominent in the thickest part of the interventricular septum near the atrioventricular junction, with some myofibers exhibiting cytoplasmic pallor and mild cytoplasmic swelling; occasional myofibers exhibited brightly eosinophilic cytoplasm and decreased cytoplasmic detail (presumptive myofiber necrosis). Smooth muscle cells within the tunica media of scattered coronary arterioles exhibited mild cytoplasmic swelling and pallor accompanied by interstitial clear spaces suggestive of mural edema (Figure 13B). Based on all available data, the primary cause of death for this bird was acute DA toxicosis. 

The adult female RTLO (07-0410, Table S1) used as a positive control for this study was found dead in Monterey County, CA in 2007 during a stranding event that coincided temporally and spatially with a *Pseudo-nitzschia* bloom with biochemically confirmed DA production. Postmortem cecal, cloacal, intestinal, and proventricular content contained up to 75,300, ng g^−1^ DA (Table S1). The loon was in good nutritional condition, with partially digested small fish (possibly anchovies), otoliths, and bones in the proventriculus and ventriculus, confirming that the bird had eaten within hours of death. The systemic veins and heart were acutely dilated and blood-filled, and marked diffuse congestion was visible in the brain and pectoral muscles. Other gross findings included moderate splenomegaly and abundant clear-red tinged pulmonary fluid and white froth.

Histopathology revealed moderate to marked diffuse congestion in the ventricular choroid plexus, lungs (Figure 11B), heart, liver, adrenal glands, and kidneys. Mild acute ventricular and perivascular hemorrhage, mild perivascular edema, and mild to moderate white matter spongiosis were also present in the brain. 

Microscopic examination of the heart revealed moderate diffuse myocardial congestion, mild epicardial hemorrhage, cytoplasmic vacuolation of myofibers, and septal edema. The tunica media of scattered coronary arterioles exhibited mild cytoplasmic swelling and pallor of mural smooth muscle cells, and possible mild intercellular edema. Marked diffuse pulmonary congestion was associated with moderate parabronchial hemorrhage and edema (Figure 11B). Based on all available data, the primary cause of death was acute DA toxicosis. 

### 2.5. Negative Controls for Acute DA Toxicosis (n = 2)

Two birds that stranded temporally and spatially unrelated to any known DA-associated event were included in the study as negative controls: an adult male PALO and an adult PALO of undetermined sex.

The adult male PALO (06-0219, Table S1) was found alive on the beach in Monterey County, California during 2006. Physical examination revealed severe emaciation, dehydration, paresis, hypothermia, reduced pupillary light responses, mild surface oiling, and severe stranding-associated pododermatitis. The bird was euthanized, and gross necropsy confirmed severe emaciation, foot trauma, and a mild ectopic mite infestation. No biological samples were collected for biochemical testing. 

Histopathology revealed diffuse muscle atrophy, serous atrophy of systemic adipose, and mild patchy perimortem rhabdomyolysis. Mild to moderate interstitial pyelonephritis and intratubular granulomas suggestive of chronic renal parasitism were also noted, although no parasites were observed. Based on all available data, the primary cause of death for this bird was euthanasia due to severe malnutrition.

The adult PALO (18-0503, Table S1) of undetermined sex stranded alive at 4-Mile Beach in Santa Cruz, California during 2018 (Table S1). Physical examination revealed that up to 25% of its body surface was contaminated with oil. Additional findings included focal dermatitis, a small oral mucosal perforation, broken feathers, and severe, bilateral, stranding-associated pododermatitis. The bird was euthanized, and gross necropsy confirmed severe pododermatitis but no grossly significant internal pathology. No biological samples were collected for biochemical testing.

Histopathology revealed mild chronic eosinophilic pyelonephritis with intralesional protozoal schizonts (renal coccidiosis), mild chronic lymphocytic typhlitis, and mild multifocal eosinophilic granulomatous enteritis of presumed parasitic origin. Based on all available data, the primary cause of death was euthanasia due to stranding-associated foot trauma.

## 3. Discussion

This is the first study to assess DA-associated avian pathology by examining wild loons with biochemically-confirmed DA toxicosis that were naturally exposed to DA and maintained in captive care for varying time periods post-exposure. Lesion assessment was achieved utilizing a suite of diagnostics including clinical observations, gross necropsy, histopathology, biochemical testing, and comparison to positive and negative controls. The core objective of this study is to better characterize the effects of DA toxicosis in birds to facilitate diagnosis. Our analysis resulted in pooling of the DA-exposed loons into three groups based on the chronicity of observed lesions: acute, subacute, and chronic. Because the subacute and chronic DA toxicosis suspects generally exhibited similar lesions with varying chronicity, these two groups were discussed together.

### 3.1. Acute DA Toxicosis Suspects and Positive Controls

Although behavioral data were unavailable for loons that were found dead due to suspected acute DA toxicosis, live stranded loons exhibited hypersalivation, obtundation, and seizures, and many died within hours [27]. In contrast to reports of DA-exposed marine mammals, laboratory animals, and other wild bird species [15,25,29,30,31], no stereotypical scratching behavior was observed. The loons and positive control birds stranded in regions with severe, extensive *Pseudo-nitzschia* blooms with high DA production, and toxin bioaccumulation was confirmed in vertebrate and invertebrate biota. Biochemical testing of necropsy samples facilitated identification of cases of acute DA toxicosis, and these test results were evaluated in relation to the observed gross and microscopic lesions. Postmortem tissues, GI content, and body fluids from positive controls and loons with suspected acute DA toxicosis had some of the highest DA concentrations ever reported in birds, humans, or marine mammals, with values as high as 681,190 ng g^−1^ (Table S1). 

At necropsy, positive controls and loons that were found dead or died early in the mass-stranding event (acute DA toxicosis group) were typically in good nutritional condition with GI tracts full of partially digested prey; findings consistent with rapid death. The most common gross lesion was diffuse, marked vascular congestion. The heart and venous circulation often had a mildly dilated or full appearance that was accompanied by marked diffuse pulmonary congestion and edema. Some cases also had acute pulmonary hemorrhage and serous fluid in the air sacs. While hypersalivation and emesis are often described in marine mammals, laboratory animals, and humans with acute DA toxicosis [21,31,32,33], emesis was not observed in the loons, although ptyalism and staining of the facial, neck, and ventral chest feathers by digesta or red-tinged fluid were observed (Figure 2A,B). Given that severe pulmonary edema was common at necropsy, the red-tinged fluid on the feathers could also represent DA-associated pulmonary hypersecretion; similar findings have been reported for sea otters with fatal acute DA toxicosis [21]. Consistent with reports for other animals affected by DA toxicosis [17,18,21], presumed collateral lesions such as blunt force trauma were also observed. Perimortem trauma can occur when incapacitated animals are tossed onto rocks or beaches by wave action, collide with other objects, or suffer from predation. 

Histopathology of the acute DA toxicosis group and positive control birds revealed diffuse, severe congestion and multifocal microhemorrhages in the brain, lateral ventricles, and heart. Neuronal and glial necrosis does not appear to be a feature of acute DA toxicosis in the avian brain, similar to reports of acute DA toxicosis in laboratory rats and sea otters [21,34]. White matter spongiosis and cardiovascular myonecrosis were relatively mild, subtle, and patchy in the acute DA toxicosis cases.

### 3.2. Subacute to Chronic DA Toxicosis Suspects

Loons that survived long enough to enter rehabilitative care often exhibited obtundation. Some also exhibited seizures and tremors, unusually severe aggression, and apparent blindness. Blindness, ocular disease, and hyperaggression have also been reported in California sea lions, sea otters, and laboratory rats with DA toxicosis [21,35,36]. As their time in care lengthened, many loons with subacute to chronic DA toxicosis exhibited chronic obtundation, somnolence, lack of a normal fear response, reduced avoidance to capture efforts, and absence of normal diving behavior, with these neurological deficits worsening over time. Some of the birds were euthanized because their clinical status plateaued and their functional deficits were considered severe enough to preclude release. 

However, even severely affected birds could appear physically normal within the artificial environment of captive care and feeding, with some birds exhibiting normal waterproofing, well-preened feathers, and reasonable nutritional and hematological parameters. Given the busy and hectic environment of wildlife rehabilitation, birds with sublethal DA toxicosis with subtle chronic behavioral deficits could easily be overlooked, released, and lost to follow-up, especially if caregivers were not familiar with normal loon behavior. Given their lack of diving activity and reduced situational and fear responses, chronically affected birds were poor candidates for post-release survival, with a high possibility of re-stranding or dying in poor nutritional condition. There was no significant evidence of these outcomes detected for the loons released after rehabilitation from this event, although the sample size was small, and federal band returns were limited. Several large-scale toxic DA blooms have occurred since the mass-stranding event described in this study; several of these toxic blooms were closely followed by mass strandings of emaciated aquatic birds [37,38,39]. Based on our research findings and temporal associations between toxic DA blooms and mass-stranding of emaciated birds, the potential for chronic cognitive deficits due to sublethal DA toxicosis in birds merits focused study. Chronic cognitive deficits are commonly reported in diverse mammalian species and humans following sublethal DA exposure [40,41].

Biochemical testing of postmortem tissues, GI content, and body fluids from loons with subacute to chronic DA toxicosis revealed surprisingly slow toxin clearance, with DA concentrations as high as 504.1 ng g^−1^ (Table S1), and birds were still DA positive up to 57 days post-stranding. Because hospitalized loons were fed biochemically confirmed, DA-free food post-stranding, this suggests that ingested toxin could be retained through tissue sequestration, enterohepatic recirculation, or other means, thereby greatly prolonging the period of toxicosis after DA ingestion. DA sequestration in fetal fluids has been confirmed in laboratory animals and California sea lions. Toxin sequestration or systemic recirculation can significantly prolong maternal and fetal exposure in mammals [42], but potential sites of DA partitioning in birds are unknown. Enterohepatic recirculation has been reported for freshwater and marine biotoxins such as microcystins and okadaic acid [43,44] but has not been described for DA, although both enteric and renal pathways are key routes for DA excretion in mammals [45].

Although DA pharmacokinetics and clearance pathways are well-understood for mammals, they are poorly characterized in birds, and key metabolic differences between these taxa could result in prolonged DA retention. For example, avian species can reflux renal output from the urodeum into the GI tract via retrograde peristalsis [46,47,48]. This post-renal mechanism helps to conserve water and electrolytes, but could potentially provide a route for reabsorption of water-soluble toxins such as DA. Another study that exemplifies these taxonomic differences demonstrated that use of allometric scaling of mammalian therapeutic data to establish antibiotic dosages in birds resulted in errors as high as 1385%, especially for antibiotics that were preferentially cleared through renal excretion [49]. Substantial differences were also observed between avian species, which suggests that various avian taxa may employ different physiochemical mechanisms to achieve xenobiotic clearance.

At gross necropsy, systemic congestion was far less prominent in the subacute to chronic DA toxicosis loons than in the acute DA toxicosis birds. Although many of the subacute DA toxicosis birds were clinically abnormal, DA-associated gross lesions were least apparent during the four-to-fifteen-day post-stranding period, which is similar to published reports for sea otters with DA toxicosis [21]. Grossly apparent encephalomalacia was first observed in the loons at 17 days post-stranding and became increasingly obvious and severe in birds that survived for longer periods of time. Chronic cases exhibited obvious pallor, thinning, and cavitation of the caudodorsal and dorsomedial cerebrum accompanied by enlargement and fluid-distention of the lateral ventricles (Figure 14B–D). Potential collateral lesions in the subacute to chronic DA toxicosis birds included aspiration pneumonia.

Histopathology revealed striking neuronal necrosis at 4 days post-stranding that was especially obvious in the caudodorsal and dorsomedial cerebrum (Figure 15A–C). Although cerebral neuroanatomy differs between birds and mammals, lesion patterns in the loon brains closely resembled descriptions of the pathophysiology of DA toxicosis in mammals. Viera et al. [34] reported delayed neuronal death, astrocytosis, and microgliosis at 5 days postexposure in laboratory rats exposed to DA and postulated that nitric oxide had a role in DA-associated neuronal degeneration.

Mild dilation of the lateral ventricles was first detected in the loons microscopically at 15 days post-stranding, becoming grossly apparent and severe in loons that survived for longer time periods. Several loons that survived 17 to 86 days post-stranding exhibited extensive cerebral encephalomalacia, thinning, and cavitation, especially in the caudodorsal and dorsomedial cerebrum. In loons that were euthanized at 57 days or more post-stranding, cavitated areas of the cerebrum were surrounded by hypercellular, gliotic neuropil with prominent vascular hypertrophy, indicative of host healing responses.

Importantly, DA-associated lesions were centered in the same anatomical areas of the brain for all loons with acute, subacute, and chronic DA toxicosis, and these lesions became larger and more chronic as the length of time post-stranding increased. Based on prior studies of avian brain anatomy [50,51], the most severely affected regions of the caudodorsal and dorsomedial cerebrum correspond to the hippocampus, hyperpallium apicale, and dorsal nidopallium, which collectively surround the lateral ventricles (Figure 15A–C and Figure 19). The brains of loons surviving 57 days or more post-stranding exhibited severe bilateral dilation of the lateral ventricles and atrophy, rarefaction and cavitation of the hippocampus, hyperpallium apicale, and dorsal nidopallium (Figure 14A–D). Subtle lesions such as spongiosis and perivascular edema were more prominent microscopically in loons with sublethal subacute to chronic DA toxicosis than in loons with acute fatal DA toxicosis. Similarly to marine mammals [17,18,21], brain lesions in loons with chronic DA toxicosis were bilateral but variably asymmetrical (Figure 14A–D and Figure 19A,B), and the marked cerebral atrophy and ventricular dilation were associated with severe clinical abnormalities. Collectively, our research findings suggest a pattern of anatomically specific, severe, chronic neurotoxicity resulting in tissue necrosis and atrophy, lesion propagation, cavitation, and secondary perilesional healing responses. This process, which presented initially as diffuse brain congestion, progressed to neuronal and glial necrosis, and ultimately resulted in severe neuropil degeneration. Because the hippocampus, hyperpallium apicale, and dorsal nidopallium surround the lateral ventricles, the observed ventricular dilation was likely accentuated by neuropil degeneration and atrophy of adjacent tissues. 

Gross or microscopic lesions of other areas of the brain of loons with DA toxicosis match lesions described in other bird species. For example, Silvagni [26] experimentally exposed pigeons (*Columba livia*) to DA via intracelomic injection and reported lesions in the cerebellar Purkinje cell layer. Although cerebellar lesions were less visually striking than lesions in other areas of the loon brain in the current study, the Purkinje cell layer was abnormal. The most consistent findings were spongiosis, swelling, cytoplasmic vacuolation, and necrosis or apoptosis of glial cells surrounding Purkinje neurons (Figure 10B–F). Henke et al. [52] reported that the molecular and Purkinje cell layers of pigeon cerebellum express kainate receptors that can serve as DA binding sites. Temporal patterns suggestive of lesion progression through time were also observed in the brain vasculature, ependyma, and choroid plexus, but were comparatively subtle [52]. 

Like to California sea lions and sea otters [19,20,21] with DA toxicosis, loons from this event, especially those that survived longer post-stranding, had gross and microscopically visible myocardial lesions; these included myo-necrosis, fatty replacement, and coronary arteriolar hyalinization which could suggest DA-mediated cardiac myofiber and arteriolar smooth muscle cell toxicity (Figure 13A–C). In humans, glutamate receptors are expressed in myocardium, blood vessels, intramural ganglia, and the conducting system, and DA toxicosis is associated with unstable blood pressure and arrhythmias [33]. Diffuse, marked vascular congestion and an acutely enlarged or “full” appearance of the heart and systemic vasculature were defining features of acute DA toxicosis in loons in this study, and in sea otters and sea lions with fatal acute DA toxicosis [19,20,21]. This suggests that DA-mediated cardiovascular disease may be important in the pathogenesis of DA toxicosis in both mammals and birds. 

In addition to direct cytotoxicity, potential indirect systemic effects can be exerted through DA-mediated damage to the CNS-based circumventricular organs, such as the area postrema [21,33,53]. Although their anatomic locations and physiology may differ, some circumventricular organs (e.g., pineal gland) are also present in avian brains [54]. The vascular architecture of these specialized structures differs from most other areas of the brain, as their capillaries lack a blood–brain barrier, unlike the typical central capillaries found in most brain tissue. This unique arrangement allows for direct exchange between circulating blood and this highly specialized nervous tissue. However, reduced shielding may make these sensory and functional interfaces between the brain and systemic circulation more vulnerable to DA-mediated cytotoxicity. The unique function of these tissues as brain/body interfaces, and their potential vulnerability to DA toxicosis may have important implications for the systemic pathophysiology of DA toxicosis in both mammals and birds; this should be a focus of future studies. 

### 3.3. Assessment and Comparison

Our analysis provides a compelling assessment of clinical signs along with gross and microscopic lesions associated with acute, subacute, and chronic DA toxicosis in birds. This effort was greatly facilitated by opportunistic sampling during a mass-stranding and mass-mortality event in 2017 that involved wild loons that were naturally exposed to DA. While this study includes loons, and systemic effects of DA toxicosis have been examined in mammals, future studies should assess the potential for systemic effects that encompass larger sample sizes and a more diverse array of avian species.

It is important to acknowledge that lesions associated with acute DA toxicosis (e.g., vascular congestion, edema, and hemorrhage) are relatively non-specific and could also result from other disease processes, such as bacterial septicemia, hyperthermia, or trauma. During the current study, each enrolled bird was assessed for both primary and any potential contributing causes of death. Evidence to support our conclusion of domoic acid as the primary cause of the observed lesions in this cohort of birds comprised the following factors: (1) Some of the highest GI and tissue DA concentrations ever reported in birds or mammals were documented in association with a well-characterized toxic bloom event and live-stranded birds with compatible neurological disease. (2) Gross necropsy and histopathology did not reveal any significant intercurrent disease processes in stranded or hospitalized birds other than stranding-associated trauma and emaciation. (3) The suspected DA lesions revealed a clear and biologically plausible temporal pattern of lesion development and propagation over time that resembled published descriptions of the pathology of acute, subacute, and chronic DA toxicosis in mammals. (4) The lesions developed and propagated within highly specific anatomic areas of the brain, heart and other tissues, as has been described for DA toxicosis in mammals. (5) Although our data are certainly preliminary, the known or hypothesized functions of key anatomic targets within the avian brain appear to correspond with observed clinical abnormalities. (6) Other than severe hippocampal atrophy, there are no pathognomonic lesions for DA toxicosis, and indicator lesions are non-specific. Diagnosis is based on a preponderance of evidence approach that employs wholistic assessment of environmental and clinical history, gross and microscopic pathology including pattern assessment in target tissues, biochemical testing of tissues and GI content for DA, and exclusion of other primary or contributing causes of death, as was performed for the current study. (7) This pilot study provides a provisional case definition based on documentation of a single severe DA-associated avian mortality event. Others should continue to test and explore our preliminary conclusions and further refine and improve this case definition across other exposure conditions and diverse avian species.

It is critical to improve diagnostics and veterinary care to accurately assess and care for birds with DA toxicosis so that impacts from increasing and intensifying blooms of *Pseudo-nitzschia* sp. with DA production can be mitigated. Given the rapid clearance of DA in other species [17,35,55,56], differences in DA concentrations observed in biological samples collected postmortem from individual seabirds was not surprising; it could be attributed to inconsistent DA exposure and bloom event timelines, and individual differences in toxin depuration. Broad variations in DA concentrations in animals from the same area have been extensively reported in the published literature, especially for shellfish and fish [57,58,59,60,61,62]. Variable DA concentrations in biological samples obtained from the same animal cohort has been reported previously in seabirds [37,39,61,62]; avian sampling is typically opportunistic, and birds encounter DA-contaminated prey at multiple trophic levels, have different prey preferences and differing habitat use, and reside within a constantly changing ecosystem. It is therefore expected that DA concentrations in seabirds exposed to DA will differ even within the same event period and location.

Definitive diagnosis of DA toxicosis remains challenging in both birds and mammals because the lesions are relatively innocuous, non-specific, and easy to confuse with artifact due to autolysis, poor or delayed fixation, and other causes. Previously published papers about natural or experimentally induced DA toxicosis in birds have not described significant DA-associated brain and cardiac lesions (Table S1). However, all of these studies assessed birds with short post-exposure intervals, and the exposure periods ended while the associated DA lesions were most subtle and non-specific. 

There are two potential explanations as to why the lesions were so severe and grossly apparent in loons from the 2017 mass-stranding event in this study, when compared to prior studies. First, biochemical testing revealed extremely high levels of DA in loons from this 2017 event, especially birds that stranded dead or died within 48 h of stranding. This suggests that the loons were exposed to higher DA concentrations than in previous studies of DA-exposed wild birds or marine mammals (Table S1). For example, cloacal content from a loon from the 2017 event (17-0086) contained DA concentrations that were 116 times higher than the LD50 reported for the cloacal content of murres experimentally exposed to DA by Silvagni [26]. Secondly, some loons from the 2017 event were in rehabilitative care for up to 12 weeks post-stranding, which allowed adequate time for the development of obvious subacute and chronic lesions. 

Factors that greatly facilitated lesion assessment for this study include (1) Availability of comparison tissues from well-characterized positive and negative control birds. (2) Comprehensive biochemical testing of GI content, tissues, and body fluids for DA, and (3) Some live-stranded loons from the 2017 mass-stranding event were in rehabilitative care for up to 86 days post-stranding, which provided our research team with the ability to detect and assess patterns of lesion progression over time. To our knowledge, no similar extended post-exposure study has been performed previously for birds exposed to DA. 

Historical accounts of DA toxicosis in seabirds have generally focused on characterizing acute behavioral abnormalities, GI content assessment, and analysis of DA in prey items that overlap temporally and spatially with seabird mortality events. Studies that assess relationships between DA biochemistry in postmortem samples and avian clinical histories, nutritional condition and gross and microscopic lesions are very limited [7,8]. For future naturally occurring, mass-stranding events associated with DA production, it would be beneficial to carefully examine birds exposed to DA to further characterize the pathophysiology of DA toxicosis using larger sample sizes, multiple avian species, and more controlled and varied time periods post-stranding. 

To date, DA-associated lesions are most extensively characterized in mammals, with key DA targets identified in the brain, heart, and other tissues. These include the hippocampus, amygdala, thalamus, ventricles, peri-hippocampal gyrus, myocardium, and systemic vasculature [17,18,21,63]. Chronic consequences to sublethal DA exposure, such as impaired spatial memory and epilepsy, have been attributed to DA-mediated hippocampal damage and disruption of hippocampal-thalamic connectivity [18,64]. 

Although our sample size is small, we observed some striking similarities between birds and mammals with DA toxicosis. For example, lesion progression over time in loons exposed to DA closely resembled that of mammals with acute, subacute, and chronic DA toxicosis [17,18,21]. The hippocampus and lateral ventricles are important targets of DA-mediated tissue damage in birds, as in mammals, along with the avian-specific hyperpallium apicale and dorsal nidopallium. Despite significant differences between avian and mammalian brains, functional homologies are being identified that could help guide future studies of DA-mediated avian neurotoxicity. For example, Atoji et al. [65] proposed homology of the dorsomedial subdivision and V-shaped layers of the avian hippocampus to the mammalian Ammon’s horn and dentate gyrus, respectively. Subsequent studies could more precisely map patterns of DA-associated avian brain toxicity, thereby further optimizing diagnostic accuracy of the effects of DA toxicosis.

As with mammals exposed to DA, our findings suggest that birds surviving sublethal DA toxicosis can suffer from chronic, potentially lifelong, cognitive impairment that could impact survival. In this study, birds with severe, chronic cognitive impairment often had grossly apparent brain lesions. Research on functional associations between observed brain lesions and clinical abnormalities would be beneficial, along with studies on the clinical efficacy of medications such as midazolam to control seizures and facilitate avian survival and recovery.

Although the spatial locations of most severe damage differed between avian and mammalian brains (in part reflective of anatomical differences between birds and mammals), the temporal sequence of lesion development, and the gross and microscopic appearance of the DA-associated avian brain lesions were strikingly similar to descriptions of severe subacute to chronic DA toxicosis in marine mammals, laboratory animals, and humans [17,21,33]. One striking advantage for diagnosing severe chronic sublethal DA toxicosis in avian brains is that highly vulnerable structures (the hippocampus, hyperpallium apicale, dorsal nidopallium and lateral ventricles) are located superficially on the caudodorsal and dorsomedial surfaces of the cerebrum, where they are easily observed during gross necropsy (Figure 14). In contrast, the mammalian hippocampus and ventricles are located deep within the cerebrum, so potential DA-associated gross lesions can only be detected after sectioning and examining formalin-fixed tissues. 

As with mammals, the avian hippocampus and hyperpallium apicale influence behaviors crucial for survival, including spatial navigation and spatial memory, stress responses, reproduction, and taxon-specific traits such as food caching [66,67,68]. Another apparent DA target is the nidopallium caudolaterale, which is a center of sensory consciousness in birds [69]. By understanding these apparent DA targets and their functions, DA-mediated damage could readily explain the clinical abnormalities displayed by loons exposed to DA, including seizures, obtundation, and abnormal fear responses. 

In this study, grossly obvious damage of the caudodorsal cerebrum surrounding the lateral ventricles suggests that these DA target sites have potential shared functional relationships and/or a common route of DA exposure, such as through penetration of DA into CSF. DA-associated lesions were also observed in other periventricular tissues within the loon brains, including the choroid plexus and ependyma, supporting the theory that proximity to ventricular spaces and CSF exposure could exacerbate DA-mediated brain toxicity. A similar pattern was described in sea otters exposed to DA [21], and DA has been detected in mammalian CSF. 

Although loons are quite distant taxonomically from other birds, research on avian species that are more well-characterized suggests mechanisms that may allow potential recovery after neuronal damage. Neurogenesis has been identified in brains of adult pigeons, especially in the hippocampus [70], while remodeling of the hippocampus and other brain regions can also occur seasonally in some avian species [71]. A study by Law et al. [72] presented evidence that induced unilateral lesions of the hippocampus in adult passerines enhanced hippocampal neurogenesis in both the injured and uninjured sides. An interesting finding in homing pigeons is that lesions of the hippocampal formation did not impede navigational map retention [73], which suggests that remodeling or neurogenesis occurred in the brain for this important hippocampal function. Adult neurogenesis and seasonal neuronal remodeling appear to provide some avian brains with enhanced neuroplasticity as compared to mammals. It is unknown whether loons or other aquatic bird species possess these potentially protective physiological capabilities, but it is a possibility worth exploring. Following this 2017 mass-stranding event, 49 loons were federally banded and released post-rehabilitation to an area north of the active toxic bloom zone (and in the direction of their interrupted northward migration). As of July 2024, only two of these birds have been encountered: a RTLO that was found dead 111 miles north of its release site 2 weeks after release and a COLO that was re-admitted for rehabilitation in Alberta, Canada, 1600 miles away, 1 month after release. The COLO was re-released following treatment for minor wounds and was not observed to be exhibiting abnormal behavior while in care. Although our resight data are limited, they suggest that birds that have recovered clinically from DA toxicosis may retain adequate navigational memory to continue migration.

## 4. Conclusions

There are several key takeaways from this multifaceted opportunistic investigation of a mass-stranding and mass-mortality event in 2017 that involved many loons naturally exposed to DA: 

### 4.1. Very High DA Concentrations and Prolonged Toxin Retention in Exposed Birds

The concentrations of DA found in postmortem tissues, body fluids, and digesta from birds in this study were some of the highest ever measured in naturally exposed wildlife. The average DA values recorded in this study were extremely high compared to prior reports in both mammals and birds. Additionally, the retention of toxin in birds undergoing rehabilitative care found in this study was much longer than any prior reports. This retention of DA in the body provided a prolonged exposure to toxin that has not previously been documented and may illustrate physiologic differences between mammalian and avian excretory mechanisms through recirculation of water-soluble toxins. 

### 4.2. Irreversible Cognitive Decline over Time in Some Survivors

Behavioral characteristics of birds with suspected acute DA toxicosis were marked by neurological abnormalities that were similar to those previously described in the literature [7,8,26]. In contrast, some birds in the suspected subacute to chronic DA toxicosis subgroup displayed behavioral abnormalities that varied over time. Because not all birds exposed to DA strand alive and come into care, the cognitive impairment described in birds in this study may be more ubiquitous than is currently documented, and these impacts may negatively influence foraging success and ultimately reduce long-term survival. However, there may be a threshold of DA exposure where healing of damaged tissues is feasible, especially given temporary supportive care. Forty-two percent of the live-captured birds affected by this event recovered sufficiently to meet release criteria, and at release were able to form into cohesive small flocks and fly away together in the correct direction of their interrupted migration. This may provide evidence of recovery of both brains and hearts. Of the birds that were released and banded, the few leg band returns that have been documented have shown post-release loons were found in locations expected during migration. 

### 4.3. Distinct Lesion Patterns and Lesion Progression over Time

Gross necropsy and microscopic analysis revealed distinct lesion patterns for birds with suspected DA toxicosis, and lesion severity corresponded with the amount of time loons survived post-stranding. Both the brain and the heart were impacted in seabirds affected by DA toxicosis. Our research findings can help to facilitate diagnosis of sublethal chronic DA toxicosis in birds. 

### 4.4. Using an Especially Severe Natural Bloom Event to Clarify Short- and Long-Term Health Impacts of DA in Aquatic Birds

This opportunistic study of loons with DA toxicosis was conducted over a longer period of time than in previous studies of HAB-related seabird stranding events or controlled laboratory experiments. The high concentration of DA exposure and longer postexposure period provided a unique opportunity to investigate the effects of DA toxicosis under natural conditions. Lesion patterns of avian DA toxicosis showed many similarities to patterns described in marine mammals, humans, and laboratory animals with DA toxicosis. This work expands our understanding of the effects of DA toxicosis in birds, people and other mammals, and re-emphasizes the value of a cohesive, “One Health” approach to wildlife research. 

## 5. Materials and Methods

### 5.1. Case Selection and Subgroup Delineation

This was an opportunistic sampling event. Examined seabirds included minimally decomposed loons that were found freshly dead during the mass-stranding event and live beachcast loons from the vicinity of the bloom area that died or were euthanized after varying lengths of time in captive care, plus positive and negative control birds collected during prior mortality events that provided reference tissues for comparison. Differences in diagnostic results, clinical, gross, and histologic pathology merited grouping the DA-exposed loons into acute, subacute, and chronic subgroups, based on the post-stranding survival period, which directly corresponded to the appearance, severity, and chronicity of observed lesions. Acute DA toxicosis suspects encompassed birds that died within 48 h of stranding (0–48 h). Birds that spent more time in rehabilitative care were considered to be subacute (4 to 15 days) or chronic cases (17–86 days). These time-based inclusion criteria aligned with distinct changes in systemic, brain, and cardiac pathology, which further aided in distinguishing the groups. Some of these lesions worsened over time in a continuum, as discussed in detail in the Results.

### 5.2. Acute DA Toxicosis Suspects (n = 10)

Loons found freshly dead on the beach during the first 2 weeks of the 2017 mass-stranding event or were in care at the International Bird Rescue’s Los Angeles Wildlife Center in San Pedro, CA (IBR) for less than 48 h exhibited biochemical, gross and microscopic similarities to each other and were considered to be acute DA toxicosis suspects. Minimally decomposed, non-scavenged, unfrozen carcasses were submitted to the California Department of Fish and Wildlife, Office of Spill Prevention and Response, Marine Wildlife Veterinary Care and Research Center in Santa Cruz, CA (CDFW) for gross necropsy and histopathology. Tissues and GI content, where available, were submitted to the University of California, Santa Cruz (UCSC) for biochemical testing to determine the presence and concentration of DA.

### 5.3. Subacute to Chronic DA Toxicosis Suspects (n = 8)

Live-stranded loons collected by local wildlife rehabilitation organizations during the 2017 mass-stranding event, transported to IBR within 2–24 h of capture, and surviving more than 48 h in care exhibited biochemical, gross and microscopic similarities to each other and were considered to be subacute (4 to 15 days postexposure) or chronic (≥17 days postexposure) DA toxicosis suspects, with live-stranded loons surviving between 4 and 86 days post-stranding. Each bird received a detailed physical examination and assessment of neurological status and physiological needs at admittance, and this information was used to guide clinical care such as addressing wounds and/or thermoregulatory, hydration, and nutritional deficits. Daily and periodic examinations were performed by experienced caregivers who recorded behavior and other clinical data throughout the rehabilitation period. Birds that were deemed unfit for release due to stranding-associated trauma, chronic behavioral abnormalities that persisted for several weeks without improvement, or other factors were humanely euthanized. Gross necropsies were performed at IBR within 2 h after death, and tissues were collected for histopathologic processing and examination at CDFW. Tissues and GI content were submitted to UCSC for biochemical testing to determine the presence and concentration of DA.

### 5.4. Positive Controls (n = 2)

Positive control seabirds were necropsied through the CDFW-OSPR Seabird Health Program prior to the 2017 mass-stranding event. Positive controls included a freshly dead PALO and a BRPE with case histories, biochemical test results, and gross and histological findings that strongly supported a diagnosis of death due to severe acute DA toxicosis. These positive controls provided reference samples for comparison with loons from the 2017 mass-stranding event. Tissues and GI samples were submitted to UCSC (15-0100) or the California Animal Health & Food Safety Laboratory (CAHFS; 07-0410) for biochemical testing to determine the presence and concentration of DA. 

### 5.5. Negative Controls (n = 3)

Negative control seabirds stranded due to factors unassociated with DA toxicity had no clinical history of abnormal behavior, and their stranding details were temporally and spatially unrelated to any known HAB events. Two live-stranded PALO and single COLO were euthanized at IBR for humane reasons, and their brains were collected in formalin within 2 h of euthanasia. Although postmortem biological samples were not submitted for biochemical testing, based on their histories these birds served as reference material to establish normal gross and microscopic avian anatomy. 

### 5.6. Biochemical Testing (Total n = 18)

Biological samples submitted for DA testing at UCSC were analyzed using an Agilent 6150 LC/MS system (Agilent Technologies, Santa Clara, CA, USA) using a Phenomenex Kinetex 2.6 µm 100 × 2.1 mm C18 column (Torrance, CA, USA). Between 0.1 and 1.5 g tissue was extracted in 50% methanol (Fisher Scientific, Waltham, MA, USA) and cleaned using Biotage ISOLUTE SAX SPE columns (Uppsala, Sweden) as described in Peacock et al. [74]. DA was quantified by peak area and retention time, using an external standard curve with NRC-Canada Certified Reference Materials (DA-g; Ottawa, Ontario, Canada). A subset of samples was analyzed after standard addition of NRC-CRM DA to confirm peak identity and to check for matrix effects. The Method Detection Limit (MDL) was 0.30 ng g^−1^ for 1 g tissue (the MDL for specific samples was dependent on sample weight). Biological samples (07-0410; positive control) submitted for biochemical testing at CAHFS were analyzed for DA presence and concentration via LC/MS using previously described methods [75].

## Data Availability

The original contributions presented in this study are included in the article/Supplementary Materials. Further inquiries can be directed to the corresponding author.

## Supplementary Materials

The following supporting information can be downloaded at: https://www.mdpi.com/article/10.3390/toxins18090401/s1, Table S1: Summarized findings for all examined birds. Results include clinical history, biochemical testing results, gross pathology, histopathology, and cause of death. Video S1: Video of PALO (17-0406) that was euthanized at 17 days post-stranding exhibited chronic behavioral abnormalities described as chronic obtundation and somnolence, “floating oddly in the pool”, not diving, and lack of avoidance to capture. Video S2: Video of PALO (17-0615) that was euthanized at 86 days post-stranding due to chronic somnolence and obtundation. Video shows bilateral lesion that appeared more severe over the left cerebral hemisphere.
