# Supplementary material for: Clinical and Pathological Findings in Coastal Birds Affected by Domoic Acid Toxicity
Source: Toxins (Basel). 2026 Sep 20;18(9):401. doi: 10.3390/toxins18090401 (PMC13611401; doi:10.3390/toxins18090401)
Supplement: Supplementary file 1 [file toxins-18-00401-s001.zip › toxins-4437138-Table S1.pdf]

**Table S1.** Summarized findings for all examined birds. Results include clinical history, biochemical testing results, gross pathology, histopathology, and cause of death.

| Case number | Pathology number | Species | Study cohort (Length of time survived post-stranding) | Clinical history | Gross photos available | GI tract contained partially digested fish postmortem | Postmortem DA concentrations ($ng\ g^{-1}$) | Gross pathology: Brain | Gross pathology: Other tissues | Histopathology: Brain | Histopathology: Other tissues | Cause of death or reason for euthanasia |
|---|---|---|---|---|---|---|---|---|---|---|---|---|
| 17-0084 (MWVCRC) | 18S0020 | PALO | Acute DA toxicosis (0 days) | Recovered dead during confirmed *Pseudonitzschia* bloom event with DA production. | Yes | Yes, suspected anchovy | Liver: 3,690.00<br>Bile: 580.00<br>Kidney: N/A<br>Cloacal contents: 6,300.00<br>Stomach contents: 6,270.00<br>Spleen: N/A | Diffusely congested brain and meninges. Meningeal venous engorgement. | Perimortem blunt trauma with hepatic and renal fragmentation. Diffusely congested lungs, liver, kidneys, and adrenals. Diffuse venous engorgement and pulmonary edema. Pododermatitis. | Diffusely congested brain and meninges. | Diffuse congestion and venous engorgement. Marked diffuse pulmonary edema. Acute hepatic rupture. | Dead on arrival. Perimortem blunt trauma, acute DA toxicosis. |
| 17-0085 (MWVCRC) | 18S0021 | RTLO | Acute DA toxicosis (0 days) | Recovered dead during confirmed *Pseudonitzschia* bloom event with DA production. | Yes | Yes, suspected anchovy | Liver: 1,390.00<br>Bile: 27,300.00<br>Kidney: N/A<br>Cloacal contents: 60,300.00<br>Stomach contents: 8,810.00<br>Spleen: N/A | Possible diffuse brain swelling. Diffusely congested brain and meninges. Meningeal venous engorgement. | Bloody fluid on feathers of ventral chest, nares, and mouth. Diffusely congested lungs, liver, kidneys, and adrenals. Diffuse venous engorgement and mild atrial dilation. Marked diffuse pulmonary edema. | Diffusely congested brain and meninges. Lymphohistiocytic and heterophilic meningoencephalitis (presumed parasitic). | Minimal endocarditis, adrenalitis, and ganglioneuritis. Focal proventricular granuloma (degenerating nematode). Intestinal cestodes. | Dead on arrival. Acute DA toxicosis, possible protozoal meningoencephalitis. |
| 17-0086 (MWVCRC) | 18S0022 | PALO | Acute DA toxicosis (0 days) | Recovered dead during confirmed *Pseudonitzschia* bloom event with DA production. | Yes | Yes, suspected anchovy | Liver: 6,850.00<br>Bile: 49,690.00<br>Kidney: N/A<br>Cloacal contents: 681,190.00<br>Stomach contents: 71,150.00<br>Spleen: N/A<br>Fish viscera in esophagus: 88,630.00 | Diffusely congested brain and meninges. Meningeal venous engorgement. | Diffusely congested lungs, liver, kidneys, skeletal muscle, and adrenals. Diffuse venous engorgement and mild atrial dilation. Marked diffuse pulmonary edema. Thin body condition. | Diffusely congested brain and meninges. Possible white matter spongiosis. Minimal ventricular hemorrhage. | Diffuse congestion, venous engorgement, and pulmonary edema. Patchy cardiac myofiber necrosis. Trace aspiration pneumonia. Pyelonephritis with intraluminal bacteria and urates. Patchy jejunitis with intralesional foreign bodies. | Dead on arrival. Acute DA toxicosis, thin body condition, bacterial pyelonephritis. |
| 17-0144 (MWVCRC)<br>17-0443 (IBR) | N/A | PALO | Acute DA toxicosis (0 days) | Recovered alive during confirmed *Pseudonitzschia* bloom event with DA production. Regurgitation and severe dehydration noted. | Yes | No | Liver: 4.13<br>Bile: 63.09<br>Kidney: 61.37<br>Cloacal contents: N/A<br>Stomach contents: N/A<br>Spleen: N/A | No abnormalities noted. | Moderate congestion in liver and lungs. Thin body condition. | N/A | N/A | Died shortly after intake to rehabilitation (IBR-LA). Acute DA toxicosis. |
| 17-0145 (MWVCRC)<br>17-0449 (IBR) | N/A | PALO | Acute DA toxicosis (0 days) | Recovered alive during confirmed *Pseudonitzschia* bloom event with DA production. Bilateral bone exposure to wing potentially due to trauma caused by neurological dysfunction. Unable to dive and paddle in water. Moderate dehydration. | Yes | No | Liver: 3.93<br>Bile: 2.82<br>Kidney: 7.95<br>Cloacal contents: N/A<br>Stomach contents: N/A<br>Spleen: N/A | No abnormalities noted. | Diffusely congested lungs, liver, kidneys, and skeletal muscle. Marked diffuse pulmonary edema. Thin body condition. | N/A | N/A | Euthanized due to injury and neurological signs including the inability to dive and paddle. Acute DA toxicosis. |
| 17-0147 (MWVCRC)<br>17-0464 (IBR) | N/A | PALO | Acute DA toxicosis (0 days) | Recovered alive during confirmed *Pseudonitzschia* bloom event with DA production. Head twitching, no reaction to human touch at intake. Moderate dehydration. | Yes | No | Liver: 3.40<br>Bile: 6.54<br>Kidney: 1.79<br>Cloacal contents: N/A<br>Stomach contents: N/A<br>Spleen: N/A | No abnormalities noted. | Diffusely congested lungs, liver, kidneys, and skeletal muscle. Emaciated. | N/A | N/A | Euthanized on arrival for neurological impairment. Acute DA toxicosis. |
| 17-0149 (MWVCRC)<br>17-0868 (IBR) | N/A | PALO | Acute DA toxicosis (0 days) | Recovered alive during confirmed *Pseudonitzschia* bloom event with DA production. Severe dehydration. Thin body condition. | Yes | No | Liver: 2.51<br>Bile: 36.43<br>Kidney: 7.84<br>Cloacal contents: N/A<br>Stomach contents: N/A<br>Spleen: N/A | Hematoma noted above skull cap. | Diffusely congested lungs, liver, kidneys, skeletal muscle, and thyroid. Diffuse venous engorgement and mild atrial dilation. Marked diffuse pulmonary edema. Thin body condition. | N/A | N/A | Died shortly after intake to rehabilitation. Acute DA toxicosis. |
| 17-0150 (MWVCRC)<br>17-0869 (IBR) | N/A | PALO | Acute DA toxicosis (0 days) | Recovered dead during confirmed *Pseudonitzschia* bloom event with DA production. | Yes | No | Liver: 1.02<br>Bile: N/A<br>Kidney: 4.10<br>Cloacal contents: N/A<br>Stomach contents: N/A<br>Spleen: N/A | No abnormalities noted. | Diffusely congested liver. Diffuse venous engorgement and mild atrial dilation. Thin body condition. | N/A | N/A | Dead on arrival. Acute DA toxicosis (presumed). |
| 17-0140 (MWVCRC)<br>17-0339 (IBR) | N/A | PALO | Acute DA toxicosis (< 48 hours) | Recovered alive during confirmed *Pseudonitzschia* bloom event with DA production. Diarrhea noted. | Yes | N/A (on captive care diet) | Liver: 0.65<br>Bile: 1,668.87<br>Kidney: 33,446.55<br>Cloacal contents: N/A<br>Stomach contents: N/A<br>Spleen: N/A | No abnormalities noted. | Moderate congestion in heart, diffusely congested liver. Large cloacal impaction and uratestasis in kidneys. | N/A | N/A | Died shortly after intake (IBR-LA). Acute DA toxicosis (presumed). |
| 17-0146 (MWVCRC)<br>17-0452 (IBR) | N/A | PALO | Acute DA toxicosis (< 48 hours) | Recovered alive during confirmed *Pseudonitzschia* bloom event with DA production. Labored breathing in care and hock luxation. Mild dehydration. | Yes | N/A (on captive care diet) | Liver: 3.82<br>Bile: 76.28<br>Kidney: 6.50<br>Cloacal contents: N/A<br>Stomach contents: N/A<br>Spleen: N/A | No abnormalities noted. | Diffusely congested lungs, liver, kidneys, skeletal muscle, and thyroid. Thin body condition. | N/A | N/A | Euthanized due to hock luxation. Acute DA toxicosis (presumed). |
| 17-0597 (IBR) | 18S0559 | PALO | Subacute DA toxicosis (4 days) | Recovered alive in area of confirmed *Pseudonitzschia* bloom event with DA production. Emaciation, chronic hypothermia, and perimortem dyspnea. | No | N/A (on captive care diet) | Liver: 21.07<br>Bile: 85.53<br>Kidney: 8.26<br>Cloacal contents: 8.52<br>Stomach contents: N/A<br>Spleen: N/A | No abnormalities noted. | Emaciation, aspiration pneumonia. Multiple small darkly pigmented granulomas in proventricular serosa. | Patchy acute cerebral neuronal and glial necrosis. Minimal hemorrhage in lateral ventricle. Possible meningeal edema, perivascular spongiosis, and ventricle dilation. | Patchy myocardial necrosis and epicardial hemorrhage. Pulmonary congestion and edema. Hyalinization of coronary arterioles. Aspiration pneumonia. Pyelonephritis (presumed parasitic). Granulomatous proventriculitis with intralesional parasite eggs. | Euthanized due to suspected DA toxicosis. Subacute DA toxicosis, aspiration pneumonia, emaciation. |
| 17-0141 (MWVCRC)<br>17-0345 (IBR) | N/A | PALO | Subacute DA toxicosis (6 days) | Recovered alive during confirmed *Pseudonitzschia* bloom event with DA production. Neurological signs, poor digestion, and no thermoregulation. | Yes | N/A (on captive care diet) | Liver: 315.05<br>Bile: 82.54<br>Kidney: 94.53<br>Cloacal contents: N/A<br>Stomach contents: N/A<br>Spleen: N/A | No abnormalities noted. | Diffusely congested liver, lungs, and kidneys. Diffuse venous engorgement. Marked diffuse pulmonary edema. Thin body condition. | N/A | N/A | Euthanized due to suspected DA toxicosis, inability to thermoregulate, and floating listlessly. Subacute DA toxicosis. |
| 17-0142 (MWVCRC)<br>17-0346 (IBR) | N/A | PALO | Subacute DA toxicosis (6 days) | Recovered alive during confirmed *Pseudonitzschia* bloom event with DA production. Clinically dehydrated with neurological signs (holding head and neck oddly, swimming oddly, not preening, and easy to catch). | Yes | N/A (on captive care diet) | Liver: < MDL<br>Bile: < MDL<br>Kidney: < MDL<br>Cloacal contents: N/A<br>Stomach contents: N/A<br>Spleen: N/A | No abnormalities noted. | Good body condition. Mild congestion noted in liver. | N/A | N/A | Euthanized due to suspected DA toxicosis, no improvement, and neurological signs did not resolve. Subacute DA toxicosis. |
| 17-0439 (IBR) | 18S0558 | RTLO | Subacute DA toxicosis (15 days) | Recovered alive in area of confirmed *Pseudonitzschia* bloom event with DA production. Chronic behavioral abnormalities (highly stressed and aggressive), stranding-related pododermatitis, and emaciation. | No | N/A (on captive care diet) | Liver: < MDL<br>Bile: < MDL<br>Kidney: < MDL<br>Cloacal contents: N/A<br>Stomach contents: N/A<br>Spleen: < MDL | No abnormalities noted. | Emaciation, pododermatitis. | Mild perivascular edema and spongiosis. Possible mild ventricular dilation and periventricular spongiosis. Mild ventricular and meningeal edema and hemorrhage. | Mild epicardial hemorrhage, cardiac myonecrosis, and hyalinization of coronary arterioles. Pulmonary hemorrhage and edema. Pyelonephritis with intracytoplasmic coccidia. | Euthanized due to chronic behavioral abnormalities, pododermatitis, and emaciation. Subacute DA toxicosis. |

**Table S1.** Summarized findings for all examined birds. Results include clinical history, biochemical testing results, gross pathology, histopathology, and cause of death.

| | | | | | | | | | | | | |
|---|---|---|---|---|---|---|---|---|---|---|---|---|
| 17-0406 (IBR) | 18S0560 | PALO | Chronic sublethal DA toxicosis (17 days) | Recovered alive in area of confirmed *Pseudonitzschia* bloom event with DA production. Chronic behavioral abnormalities (floating oddly, obtunded, somnolent, unusually easy to catch). | No (video only) | N/A (on captive care diet) | Liver: 71.71<br>Bile: 31.30<br>Kidney: 46.95<br>Cloacal contents: 504.10<br>Stomach contents: N/A<br>Spleen: < MDL | Brain abnormally soft. | No abnormalities noted. | Patchy spongiosis and gliosis. Severe bilateral cerebral encephalomalacia and cavitation. Meningeal and perivascular edema. Meningeal and ventricular hemorrhage. Ventricular dilation and ependymal regeneration. | Minimal epicardial hemorrhage, myocardial necrosis and atrophy, and coronary arteriolar hyalinization. Pyelonephritis with intracytoplasmic coccidia. Mild eosinophilic proventriculitis. Sparse intramuscular sarcocysts. | Euthanized due to chronic behavioral abnormalities. Chronic sublethal DA toxicosis. |
| 17-0353 (IBR) | 18S0557 | RTLO | Chronic sublethal DA toxicosis (18 days) | Recovered alive in area of confirmed *Pseudonitzschia* bloom event with DA production. Chronic behavioral abnormalities (described as "crazy diver") and failure to gain weight. Conjunctivitis also noted. | No | N/A (on captive care diet) | Liver: < MDL<br>Bile: N/A<br>Kidney: < MDL<br>Cloacal contents: < MDL<br>Stomach contents: N/A<br>Spleen: 129.11 | No abnormalities noted. | Thin body condition. | Meningeal, perivascular and periventricular edema, spongiosis, gliosis, and hemorrhage. Ventricular dilation. Ependymal swelling, vacuolation, and regeneration. | Mild epicardial hemorrhage. Moderate cardiac myonecrosis, fatty replacement, and coronary arteriolar hyalinization. Pulmonary hemorrhage and edema. Pectoral rhabdomyolysis and fibrosis. Pyelonephritis with intralesional coccidia and trematodes. | Euthanized due to chronic behavioral abnormalities and failure to gain weight. Chronic sublethal DA toxicosis, thin body condition. |
| 17-0316 (IBR) | N/A | PALO | Chronic sublethal DA toxicosis (57 days) | Recovered alive in area of confirmed *Pseudonitzschia* bloom event with DA production. Persistent hypothermia, chronic behavioral abnormalities (episodic somnolence and aggression, crashing into objects, not diving, abnormally easy to catch), and avian poxvirus infection. | Yes | N/A (on captive care diet) | Liver: 55.09<br>Bile: N/A<br>Kidney: 230.07<br>Cloacal contents: N/A<br>Stomach contents: N/A<br>Spleen: 142.99 | Grossly apparent, bilateral, well-demarcated zones of caudodorsal and dorsomedial cerebral pallor and atrophy, and severe ventricle dilation. Suspected diffuse cerebellar swelling. | Thin body condition. Possible mild orange-white mottling of myocardium. Historical avian poxvirus pododermatitis (removed antemortem as biopsy). | *N/A | *N/A | Euthanized due to persistent hypothermia, chronic behavioral abnormalities, and avian poxvirus infection. Chronic sublethal DA toxicosis, thin body condition. |
| 17-0615 (IBR) | 18S0561 | PALO | Chronic sublethal DA toxicosis (86 days) | Recovered alive in area of confirmed *Pseudonitzschia* bloom event with DA production. Chronic behavioral abnormalities (minimal diving, constantly floating on surface and abnormally easy to catch). Mild conjunctivitis and emaciation. | Yes (and video) | N/A (on captive care diet) | Liver: < MDL<br>Bile: < MDL<br>Kidney: < MDL<br>Cloacal contents: N/A<br>Stomach contents: N/A<br>Spleen: < MDL | Grossly apparent, bilateral but asymmetrical, well-demarcated zones of caudodorsal and dorsomedial cerebral pallor and atrophy, and severe ventricle dilation. Suspected diffuse cerebellar swelling. | Emaciation. | **Marked dilation of lateral ventricles and atrophy, rarefaction, and cavitation of adjacent neuropil. | **Heart: Mild myocardial hemorrhage, edema, and myofiber atrophy. Mild pyelonephritis with intralesional trematodes. Patchy colitis and typhlitis with intralesional parasites. | Euthanized due to chronic behavioral abnormalities. Chronic sublethal DA toxicosis, emaciation. |
| 15-0100 (MWVCRC) | 15S0558 | BRPE | Positive control (0 days) | Recovered dead during confirmed *Pseudonitzschia* bloom event with high DA production. Concurrent mass-mortality of strongly DA-positive, *Pseudonitzschia* frustule-positive anchovies. | Yes | Yes, anchovy | Cloacal contents: 285,000.00<br>Stomach contents: 160,000.00<br>Pericardial fluid 11,000.00 | Possible mild diffuse brain swelling. Diffusely congested brain and meninges and suspected meningeal hemorrhage. Meningeal venous engorgement. | Diffusely congested lungs, liver, kidneys, and adrenals. Diffuse venous engorgement, acute cardiomegaly and serous pericardial effusion. Severe pulmonary edema and red-tinged serous fluid in air sacs. | Diffusely congested brain and meninges, and patchy white matter vacuolation and edema. Minimal perivascular cuffing of venules and capillaries. | Diffuse congestion. Patchy cardiac myonecrosis, hemorrhage, and pulmonary edema. Pyelonephritis and cloacitis with intralesional trematode eggs. Mild enteritis and intralumenal trematode. | Dead on arrival. Acute DA toxicosis. |
| 07-0410 (MWVCRC) | 07S0229 | RTLO | Positive control (0 days) | Recovered dead during confirmed *Pseudonitzschia* bloom event with DA production. | External only | Yes, anchovy | Cecal contents: 75,300.00<br>Cloacal contents: 46,100.00<br>Stomach contents: 4,530.00<br>Intestinal contents: 14,600.00 | Diffusely congested brain and meninges. Meningeal venous engorgement. | Diffusely congested lungs, heart, liver, kidneys, skeletal muscles, and adrenals. Diffuse venous engorgement and acute cardiomegaly. Severe pulmonary edema. Serous pericardial effusion. Splenomegaly. | Diffusely congested brain and meninges, and mild acute ventricular hemorrhage. Perivascular edema, spongiosis, and scant acute hemorrhage. | Diffuse congestion. Pulmonary edema and hemorrhage. Severe choroidal congestion and scant ocular hemorrhage. Mild pyelonephritis, esophagitis, and typhlitis (parasitism or foreign body). | Dead on arrival. Acute DA toxicosis. |
| 06-0219 (MWVCRC)<br>06-0280 (MSPCA) | 06S0687 | PALO | Negative control (30 days) | Recovered alive. No behavioral abnormalities noted. | External only | N/A (on captive care diet) | ***Not done | No abnormalities noted. | Emaciation. | No abnormalities noted. | Mild chronic proventriculitis and pyelonephritis (suspected parasitism). Focal mild coronary arterial mural hyalinization and myofiber atrophy, ventricular septum. | Euthanized due to failure to thrive. Emaciation. |
| 18-0503 (MWVCRC)<br>18-0249 (IBR) | 18S0925 | PALO | Negative control (32 days) | Recovered alive. Mild to moderate external oiling. Stranding-associated pododermatitis. | No | N/A (on captive care diet) | ***Not done | No abnormalities noted. | Pododermatitis. | No abnormalities noted. | Eosinophilic pyelonephritis with intralesional protozoal schizonts (renal coccidiosis). Mild multifocal enteritis and typhlitis (suspected parasitism). | Euthanized due to stranding-associated pododermatitis. External oiling. |
| 23-1238 (IBR) | 23S0461 | COLO | Negative control (2 days) | Recovered alive. Moderate external oiling with associated lesions. Moderate dehydration. Toe lesions. No thermoregulation. | No | N/A (on captive care diet) | ***Not done | No abnormalities noted. | Toe lesions. Emaciation. | No abnormalities noted. Used as a clean negative control for comparisons and scanning of whole brain sections used in Figure 4. Brain only received for histopathology. | N/A | Euthanized due to lesions associated with external oiling. Emaciation. |

MDL = Method Detection Limit.
* Tissues collected postmortem for histopathology were misplaced.
** Tissues collected for histopathology were initially fixed in a fluid other than formalin, limiting precision of assessment.
*** No tissues were collected for toxicological testing.
